# Branched-Chain Aminotransferases Control TORC1 Signaling in *Saccharomyces cerevisiae*

**DOI:** 10.1371/journal.pgen.1005714

**Published:** 2015-12-11

**Authors:** Joanne M. Kingsbury, Neelam D. Sen, Maria E. Cardenas

**Affiliations:** Department of Molecular Genetics and Microbiology, Duke University Medical Center, Durham, North Carolina, United States of America; The University of North Carolina at Chapel Hill, UNITED STATES

## Abstract

The conserved target of rapamycin complex 1 (TORC1) integrates nutrient signals to orchestrate cell growth and proliferation. Leucine availability is conveyed to control TORC1 activity via the leu-tRNA synthetase/EGOC-GTPase module in yeast and mammals, but the mechanisms sensing leucine remain only partially understood. We show here that both leucine and its α-ketoacid metabolite, α-ketoisocaproate, effectively activate the yeast TORC1 kinase via both EGOC GTPase-dependent and -independent mechanisms. Leucine and α-ketoisocaproate are interconverted by ubiquitous branched-chain aminotransferases (BCAT), which in yeast are represented by the mitochondrial and cytosolic enzymes Bat1 and Bat2, respectively. BCAT yeast mutants exhibit severely compromised TORC1 activity, which is partially restored by expression of Bat1 active site mutants, implicating both catalytic and structural roles of BCATs in TORC1 control. We find that Bat1 interacts with branched-chain amino acid metabolic enzymes and, in a leucine-dependent fashion, with the tricarboxylic acid (TCA)-cycle enzyme aconitase. BCAT mutation perturbed TCA-cycle intermediate levels, consistent with a TCA-cycle block, and resulted in low ATP levels, activation of AMPK, and TORC1 inhibition. We propose the biosynthetic capacity of BCAT and its role in forming multicomplex metabolons connecting branched-chain amino acids and TCA-cycle metabolism governs TCA-cycle flux to activate TORC1 signaling. Because mammalian mitochondrial BCAT is known to form a supramolecular branched-chain α-keto acid dehydrogenase enzyme complex that links leucine metabolism to the TCA-cycle, these findings establish a precedent for understanding TORC1 signaling in mammals.

## Author Summary

In all organisms from yeasts to mammals the target of rapamycin TORC1 pathway controls growth in response to nutrients such as leucine, but the leucine sensing mechanisms are only partially characterized. We show that both leucine and its α-ketoacid metabolite, α-ketoisocaproate, are similarly capable of activating TORC1 kinase via EGOC GTPase-dependent and -independent mechanisms. Activation of TORC1 by leucine or α-ketoisocaproate is only partially mediated via EGOC-GTPase. Leucine and α-ketoisocaproate are interconverted by ubiquitous branched-chain aminotransferases (BCAT). Disruption of BCAT caused reduced TORC1 activity, which was partially restored by expression of BCAT active site mutants, arguing for both structural and catalytic roles of BCAT in TORC1 control. We find BCAT interacts with several branched-chain amino acid metabolic enzymes, and in a leucine-dependent fashion with the tricarboxylic acid (TCA)-cycle enzyme aconitase. Both aconitase mutation or TCA-cycle inhibition impaired TORC1 activity. Mutation of BCAT resulted in a TCA-cycle intermediate profile consistent with a TCA-cycle block, low ATP levels, activation of AMPK, and TORC1 inhibition. Our results suggest a model whereby BCAT coordinates leucine and TCA cycle metabolism to control TORC1 signaling. Taken together, our findings forge key insights into how the TORC1 signaling cascade senses nutrients to control cell growth.

## Introduction

The Target of Rapamycin Complex 1 (TORC1) is functionally and structurally conserved throughout eukaryotes and senses and responds to nutrients to promote cell growth and inhibit catabolic processes such as autophagy. Amino acids, particularly the branched-chain amino acid leucine, control TORC1 activity by affecting the nucleotide binding status of the Exit from G_0_ Complex (EGOC) GTPase subunits Gtr1 and Gtr2 in *Saccharomyces cerevisiae* (or the Rag GTPases in mammalian cells) [1–3]. Under leucine-starvation conditions, the yeast SEA (Seh1-associated) complex and its mammalian ortholog GATOR activate the GTPase activity of, and thereby inhibit, the EGO and Rag GTPase complexes [4–6]. Conversely, the EGO and Rag GTPase complexes are positively regulated in leucine-replete conditions by leucyl-tRNA synthetase (LeuRS) in yeast and mammals (although this model has been contested in mammals [7]), and the vacuolar ATPase in mammalian cells [8–10]. Amongst other TORC1-stimulating amino acids, arginine abundance in mammalian cells is proposed to be sensed by the lysosomal transporter SLC38A9 and conveyed to mTORC1 via the Rag GTPases [11, 12], while glutamine levels appear to be transduced to control both yeast and mammalian TORC1 independently of the EGO/Rag GTPases [13, 14]. In turn, activation of TORC1 controls yeast growth via phosphorylation of three major effector branches: activation of ribosome biogenesis via the protein kinase Sch9, and repression of autophagy, nitrogen, and stress responses via Atg13/Atg1 and Tap42-PP2A [15–19]. Furthermore, ammonium starvation, heat, oxidative, and osmotic stresses, and also low levels of carbon, phosphate, and energy, control yeast TORC1 activity by additional mechanisms involving Rho1, the AMP-regulated, MAPK, PAS, and Hog1 kinases, and stress granule sequestration [20–23].

Despite considerable focus, the mechanisms by which leucine and other nutrient signals are transduced to control TORC1 activity remain incompletely defined. Plausible mechanisms through which leucine could be sensed are as follows. First, the leucine signal may be elicited via an enzyme for which leucine is a substrate. Candidates in yeast include: 1) the key controllers of leucine metabolism, the BCATs [24]; 2) amino acid transporters, of which significant precedent already exists for acting as signal transducers [11, 12, 25–27]; 3) LeuRS, already implicated in TORC1 control [8, 9]; and 4) importantly, amino acid limitation is in general sensed through the binding of uncharged tRNAs to the Gcn2 kinase which, in conjunction with Gcn2 dephosphorylation, contributes to Gcn2 activation. However, because inactivation of TORC1 with rapamycin contributes to Gcn2 dephosphorylation, the current model is that TORC1 negatively controls Gcn2 and there is no evidence that the Gcn2 amino acid sensing mechanism impinges upon TORC1 signaling [28]. Second, leucine may serve as an allosteric activator or inhibitor of an enzyme that directly or indirectly controls TORC1. In yeast, leucine feedback-inhibits α-isopropylmalate synthase (Leu4), thereby controlling pathway flux and reducing α-isopropylmalate levels, which in turn downregulates the expression of a set of genes regulated by the Leu3-α-isopropylmalate complex [24]. Significantly, leucine is an allosteric activator of mammalian glutamate dehydrogenase (Gdh1), important for driving mTORC1 activity via glutaminolysis [29]. Third, leucine could be metabolized to a signaling compound. Leucine is metabolized by BCATs to KIC, which has been shown to support mTORC1 activity [30–33]. In yeast KIC is further metabolized to fusel alcohols that can serve as signaling molecules [34–37].

Here we investigated roles for leucine metabolites and metabolic enzymes in the control of TORC1 activity. We show that KIC is capable of stimulating TORC1 activity following leucine starvation. TORC1 stimulation by leucine or KIC is only partially reduced by leucyl-tRNA synthetase inhibition or EGOC disruption, suggesting EGOC-independent and -dependent routes of TORC1 regulation. Our studies indicate that the BCATs Bat1 and Bat2 are critical to activate TORC1 signaling. We provide evidence that Bat1 governs TCA-cycle flux via both its enzymatic activity and by signaling leucine and KIC availability through formation of a supramolecular complex with the key TCA-cycle enzyme Aco1. A mammalian BCAT-GDH metabolon is known to connect α -ketoglutarate and glutamate production that could fuel the TCA-cycle [38, 39], and thus our findings establish a foundation for understanding control of mTORC1 signaling by metabolism.

## Results

### BCATs control activation of TORC1 in response to leucine and KIC availability

**Fig 1:**
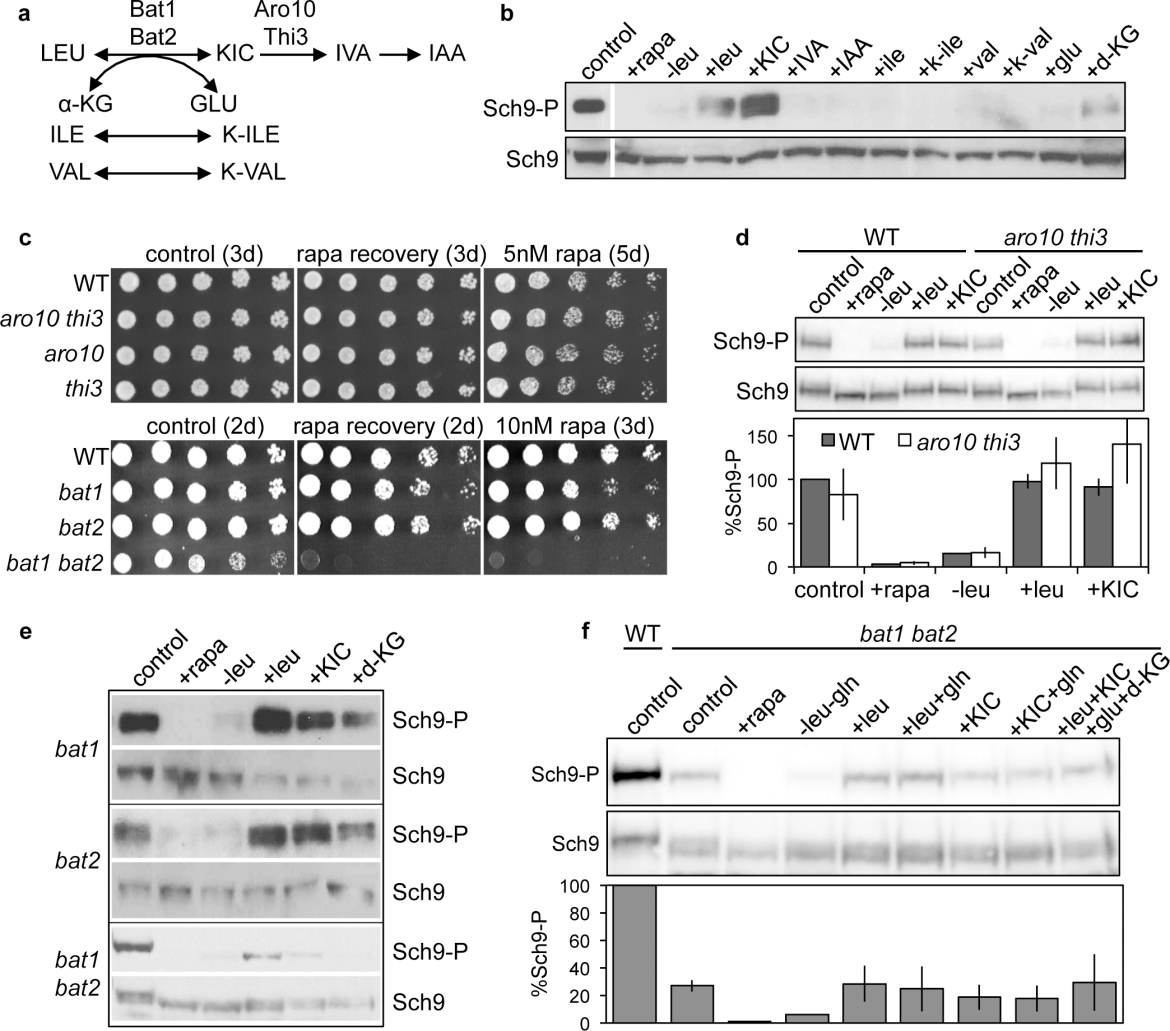
BCATs and KIC activate TORC1 signaling. (**a**) Branched-chain amino acid metabolism in *S*. *cerevisiae*. (**b**) TORC1 kinase activity of WT cells is stimulated by addition of leucine, α-ketoisocaproate (KIC), or dimethyl α-ketoglutarate (d-KG) following leucine starvation. Cells were grown in SC-his-ura-lys+gln to OD_600nm_~1, and protein extracts were prepared from the following conditions: no treatment (control), incubation with 200 nM rapamycin for 30 min (+rapa), 2 hr leucine starvation, or addition of 2 mM leucine, KIC, isovaleraldehyde (IVA), isoamyl alcohol (IAA), isoleucine, α-keto-β-methylvalerate (K-ile), valine, α-ketoisovalerate (K-val), glutamate, or d-KG, for 1 hr to cells that have been leucine-starved for 2 hr. (**b**, **d**-**f**) TORC1 activity was assessed by monitoring the phosphorylation status of Sch9 Thr737 and the overall protein levels of Sch9 by Western blot with the anti-phospho-Thr737-Sch9 (Sch9-P) and anti-732-743-Sch9 (Sch9) antibodies, respectively. Where shown, Sch9 phosphorylation was normalized to Sch9 levels and expressed as an average percentage of WT Sch9 phosphorylation from three independent experiments, with error bars depicting the standard deviation from the mean. (**c**) *bat1 bat2* mutant strains are rapamycin (rapa) sensitive and recover poorly from rapamycin-induced growth arrest. Strains were grown to OD_600nm_ ~1 in SC-his-ura-lys+gln (top panel) or YPD (bottom panel). To test recovery from rapamycin-induced growth arrest, strains were incubated for 6 hr with 200 nM rapamycin, washed, serially diluted 5-fold and plated on drug-free media as indicated. Aliquots of untreated cultures were similarly diluted and plated to media +/- rapamycin. Plates were incubated for 2 to 5 days as indicated. (**d**) TORC1 kinase activity is not reduced by *aro10 thi3* mutation. (**e**) TORC1 activity is reduced in *bat1 bat2* double mutants compared with *bat1* or *bat2* single mutants. (**f**) Leucine or KIC addition to leucine-starved *bat1 bat2* mutants stimulates TORC1 activity, although at a reduced level compared to WT. All experiments were performed in triplicate except (**b**), (**e**), and (**f**), which were performed in duplicate.

To elucidate the mechanisms via which leucine controls TORC1 activity, we investigated if any products of branched-chain amino acid (BCAA) metabolism (**Fig 1A**) are capable of stimulating TORC1 activity following leucine starvation (**Fig 1B**). Surprisingly, we found that the leucine α-ketoacid KIC was as effective as leucine in activating TORC1 (**Fig 1B**). The other BCAAs isoleucine and valine, and their respective α-ketoacids, α-keto-β-methylvalerate and α-ketoisovalerate, all failed to stimulate TORC1 activity, as monitored by phosphorylation of Sch9-Thr737. Leucine is reversibly metabolized to KIC via the BCATs Bat1 (mitochondrial) and Bat2 (cytoplasmic), in a reaction coupled to the transamination of α-ketoglutarate to glutamate (**Fig 1A**). We observed very modest TORC1 stimulation by dimethyl α-ketoglutarate (d-KG, a membrane-permeable derivative of α-ketoglutarate), but not by glutamate addition. KIC is further metabolized via Thi3/Aro10 to isovaleraldehyde (IVA) and isoamyl alcohol (IAA), both of which also failed to stimulate TORC1 activity. Furthermore, *thi3 aro10* mutants were not rapamycin hypersensitive (**Fig 1C**) and showed wild-type levels of TORC1 activity (**Fig 1D**). Therefore, conversion of KIC to IVA or IAA was not required for stimulation of TORC1 activity.

To determine if KIC stimulation of TORC1 following leucine starvation is biologically relevant and not merely due to transamination back to leucine, we tested the ability of KIC to stimulate TORC1 activity in *bat1 bat2* mutants. Mutation of *BAT1* or *BAT2* individually did not perturb either TORC1 activity or the ability of KIC and leucine to stimulate TORC1 activity following leucine starvation (**Fig 1E**). In contrast, we observed a striking ~73% reduction in TORC1 activity in the *bat1 bat2* mutant compared to the WT (**Fig 1E and 1F**). However, leucine and KIC were still similarly effective in stimulating TORC1 (albeit to reduced levels; 20–30% of the level observed in the WT strain) in the *bat1 bat2* mutant (**Fig 1E and 1F**). Thus the ability of KIC to stimulate TORC1 is, in part, unrelated to its conversion back to leucine. When combinations of leucine or KIC with glutamine, d-KG, or glutamate were added to leucine-starved cells they did not stimulate TORC1 activity above KIC or leucine addition alone, and in contrast to the WT, no stimulation by d-KG was observed (**Fig 1E**). Moreover, we found that the *bat1 bat2* mutant was hypersensitive to rapamycin and recovered less well from rapamycin-induced growth arrest than the WT and *bat1* or *bat2* single mutants (**Fig 1C**). Taken together, these results support a role for BCATs in promoting robust TORC1 activity.

### BCAT and KIC control TORC1 activity in part via the leucyl-tRNA synthetase-EGOC module

**Fig 2:**
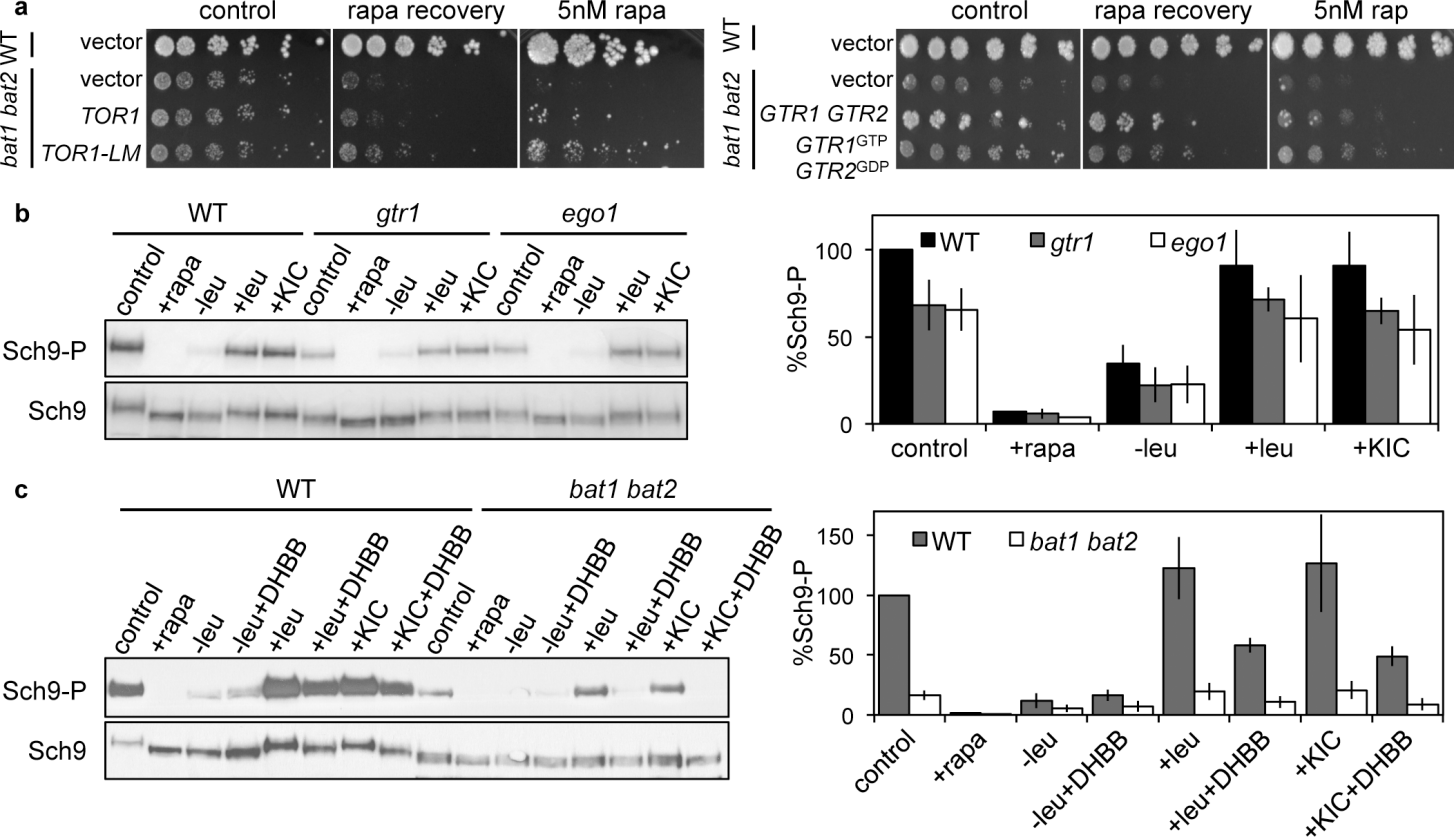
The branched-chain aminotransferases control TORC1 activity via EGOC- and leuRS-dependent and independent mechanisms. (**a**) Expression of hyperactive *TOR1-LM* and *GTR1* ^*GTP*^ *GTR2* ^*GDP*^-locked alleles partially recovered *bat1 bat2* rapamycin sensitivity and recovery defects. WT and *bat1 bat2* isogenic strains expressing the indicated *TOR1* alleles were cultured in SD+ile+val+leu+gln, and strains expressing *GTR1* and *GTR2* alleles were cultured in SC-ura-his-lys+gln, and assayed for rapamycin sensitivity and recovery as described in **Fig 1C.** Plates were incubated for 3 or 4 days (controls), 4 days (rapa recovery) and 5 or 8 days (5 nM rapa). (**b**) TORC1 kinase activity in response to leucine and KIC addition following leucine starvation was similarly reduced in *gtr1* and *ego1* mutants compared with the WT. (**c**) Leucine- and KIC-mediated stimulation of TORC1 activity following leucine starvation was inhibited by the leucine tRNA synthetase inhibitor DHBB treatment (10 μM DHBB was added 30 min prior to leu and KIC addition to leucine-starved cells) in both WT and *bat1 bat2* strains. (**b-c**) Sch9 phosphorylation was determined and quantified as described in **Fig 1**. All experiments were performed in triplicate with similar results.

To further substantiate a role for BCATs in control of TORC1 activity, we investigated whether the *TOR1*-*LM* allele, which contains a mutation in the kinase domain (Tor1^L2134M^) rendering TORC1 independent of upstream activation [23], would suppress *bat1 bat2* TORC1-signaling defects. Expression of *TOR1*-*LM* partially suppressed the rapamycin hypersensitivity and defects in recovery from rapamycin-mediated growth arrest of the *bat1 bat2* mutants (**Fig 2A**). Thus, these results support a role for BCAT upstream of TORC1.

We also investigated if BCAT control and KIC stimulation of TORC1 activity requires EGOC. Expression of *GTR1* ^*GTP*^ *GTR*2^GDP^-locked alleles that activate TORC1 independent of leucine levels partially suppressed the rapamycin hypersensitivity and recovery defects of *bat1 bat2* mutants (**Fig 2A**). Stimulation of Sch9 phosphorylation by KIC and leucine addition to leucine-starved cells was similarly only partially reduced by *gtr1* or *ego1* mutations (**Fig 2B**). These results support that the BCATs and their metabolites leucine and KIC control and stimulate TORC1 activity via EGOC and EGOC-independent inputs.

The editing activity of leucyl-tRNA synthetase (LeuRS) is implicated in sensing and signaling leucine availability to TORC1 in yeast, acting upstream of the EGOC [8]. Exposure of leucine-starved WT cells to the LeuRS inhibitor DHBB, which traps tRNA-LEU in the LeuRS editing site [8], reduced both KIC and leucine stimulation of TORC1 activity at comparable levels (**Fig 2C**). These results support that similar to leucine, KIC stimulates TORC1 activity partially via the LeuRS. Remarkably, the reduced stimulation of TORC1 activity elicited by leucine and KIC addition to leucine-starved *bat1 bat2* cells was nearly blocked by DHBB (**Fig 2C**). These findings support a model in which both BCATs and the LeuRS-EGOC module contribute independently to activate TORC1.

### BCAT mutation perturbs TORC1 activity independently from BCAA auxotrophy or biosynthetic intermediate accumulation effects

In our experiments, leucine starvation is achieved via a *leu2* mutation and incubation in media lacking leucine. Under these conditions cells accumulate the intermediate 3-isopropylmalate (3-IPM), which is subsequently converted to 3-IPM methyl ester, an invasive growth signaling molecule [40]. However, 3-IPM accumulation does not influence TORC1 activity because the TORC1 activity response to leucine starvation and leucine or KIC readdition was similar in *leu4 leu9*, *leu1*, or *leu2* mutants, which each accumulate different pathway intermediates (**S1 Fig**).

**Fig 3:**
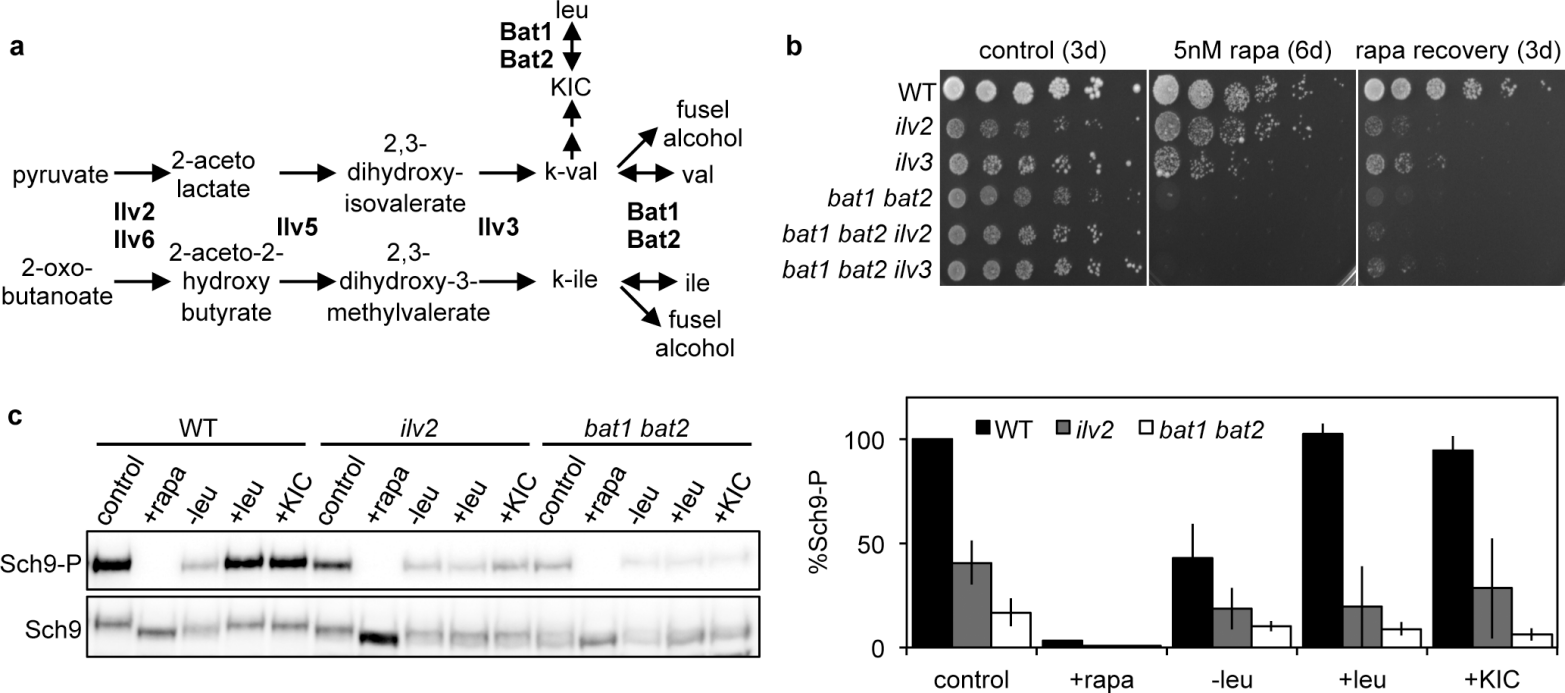
Isoleucine and valine auxotrophy contribute only partially to BCAT mutant defects in TORC1 signaling. (**a**) Isoleucine and valine biosynthetic pathway. (**b-c**) Strains disrupted for *ilv2* and *ilv3* displayed less severe defects in (**b**) rapamycin sensitivity and recovery (determined as in **Fig 1C**) or (**c**) TORC1 activity compared with the *bat1 bat2* strain. (**c**) Sch9 phosphorylation was determined in triplicate and quantified as described in **Fig 1**.

Isoleucine and valine auxotrophy, or elevation of the respective isoleucine and valine α -ketoacids and their fusel alcohol degradation products (**Fig 3A**) caused by BCAT mutation, could potentially affect TORC1 signaling. We found that *ilv2* and *ilv3* mutants, which are BCAA auxotrophs, were rapamycin-sensitive, but less hypersensitive than *bat1 bat2* mutants (**Fig 3B**). Similarly, TORC1 activity was reduced in *ilv2* mutants, but not to the low level observed for *bat1 bat2* mutants (**Fig 3C**). Furthermore, *ilv2 bat1 bat2*, and *ilv3 bat1 bat2* triple mutants, which cannot accumulate isoleucine and valine α-ketoacids and their degradation products, were as rapamycin-hypersensitive as *bat1 bat2* double mutants (**Fig 3B**). These results support that the effects of BCAT mutation in perturbing TORC1 activity are not mediated by BCAA intermediate accumulation in the *bat1 bat2* mutant, and that there are both BCAA biosynthesis-dependent and -independent roles for BCATs in TORC1 signaling.

### Bat1 interacts with TCA-cycle enzymes and BCAT mutants exhibit perturbed levels of TCA-cycle metabolites

**Fig 4:**
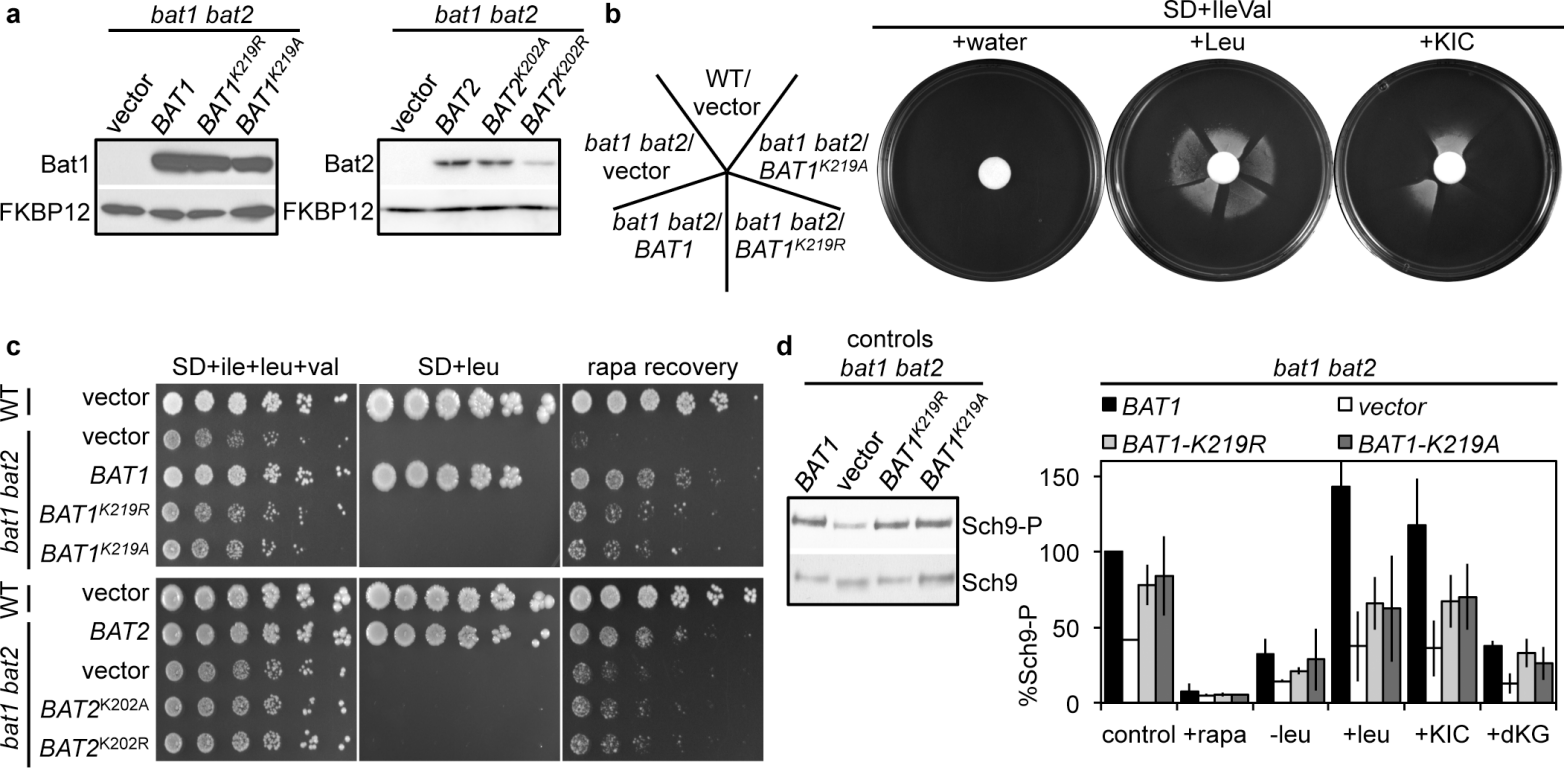
Bat1 catalytic activity only partially contributes to the Bat1 effects on TORC1 activity. (**a**) Pyridoxal binding mutant Bat1^K219R^ and Bat1^K219A^ were stably expressed in *bat1 bat2* mutants compared with WT Bat1. Western blots of protein extracts were immunoblotted using antibodies specific to *S*. *cerevisiae* BCATs and to FKBP12 (as a loading control). (**b**) KIC supplemented the leucine auxotrophy of WT and *bat1 bat2* mutants episomally expressing *BAT1*, but not *BAT1* ^K219R/A^ or the empty vector. Cultures of the indicated strains were diluted to OD_600nm_~1 and evenly applied to SD+ile+val plates using a sterile cotton swab. Water (50 μl), leucine (7.6 μM) or KIC (10 μM) were then added to a 12 mm filter disc placed in the center of the plate. Plates were photographed after 3 d incubation. (**c**) Expression of *BAT1* and *BAT2* active site mutants (*BAT1* ^*K219R/A*^ and *BAT2* ^*K202R/A*^) failed to alleviate the *bat1 bat2* mutant ile and val auxotrophy (SD+leu plates). Note that all strains are also *leu2* mutants and are therefore auxotrophic for leucine regardless of BCAT activity. *BAT1* ^*K219R*^ or *BAT1* ^*K219A*^ expression partially restored rapamycin recovery defects of *bat1 bat2* mutants compared with the empty vector control. (**d**) TORC1 kinase activity of *bat1 bat2* strains expressing *BAT1*, *BAT1* ^*K219A*^ or *BAT1* ^*K219R*^. Sch9 phosphorylation was determined in triplicate as described in **Fig 1D** and normalized against activity from strains expressing *BAT1* grown in the control condition.

To further test whether a BCAT enzymatic role is required to activate TORC1 signaling, we mutated the conserved pyridoxal phosphate-binding site [41] of *BAT1* (*BAT1* ^*K219R/A*^) and *BAT2* (*BAT2* ^*K202R/A*^) to eliminate BCAT activity. *BAT1* ^*K219R/A*^ and *BAT2* ^*K202A*^ were as stably expressed as the WT *BAT1* and *BAT2* respectively (although *BAT2* ^*K202R*^ was less well expressed) (**Fig 4A**). Loss of BCAT activity of plasmid-expressed *BAT1* ^*K219R/A*^ was confirmed by an inability to utilize KIC to supplement the leucine auxotrophy (**Fig 4B**), and an inability to complement the *bat1 bat2* strain leucine, isoleucine and valine auxotrophy (**Fig 4C**). Surprisingly, the *BAT1* ^*K219R/A*^ alleles, but not the equivalent *BAT2* ^*K202R/A*^ alleles, were nearly as effective as WT *BAT1* or *BAT2* at partially suppressing the rapamycin recovery and TORC1 activity defects of the *bat1 bat2* mutant (**Fig 4C and 4D**). Taken together, these results suggest that both Bat1 and Bat2 contribute to TORC1 signaling, likely through their BCAT biosynthetic role, and that in addition Bat1 plays a non-enzymatic role.

**Fig 5:**
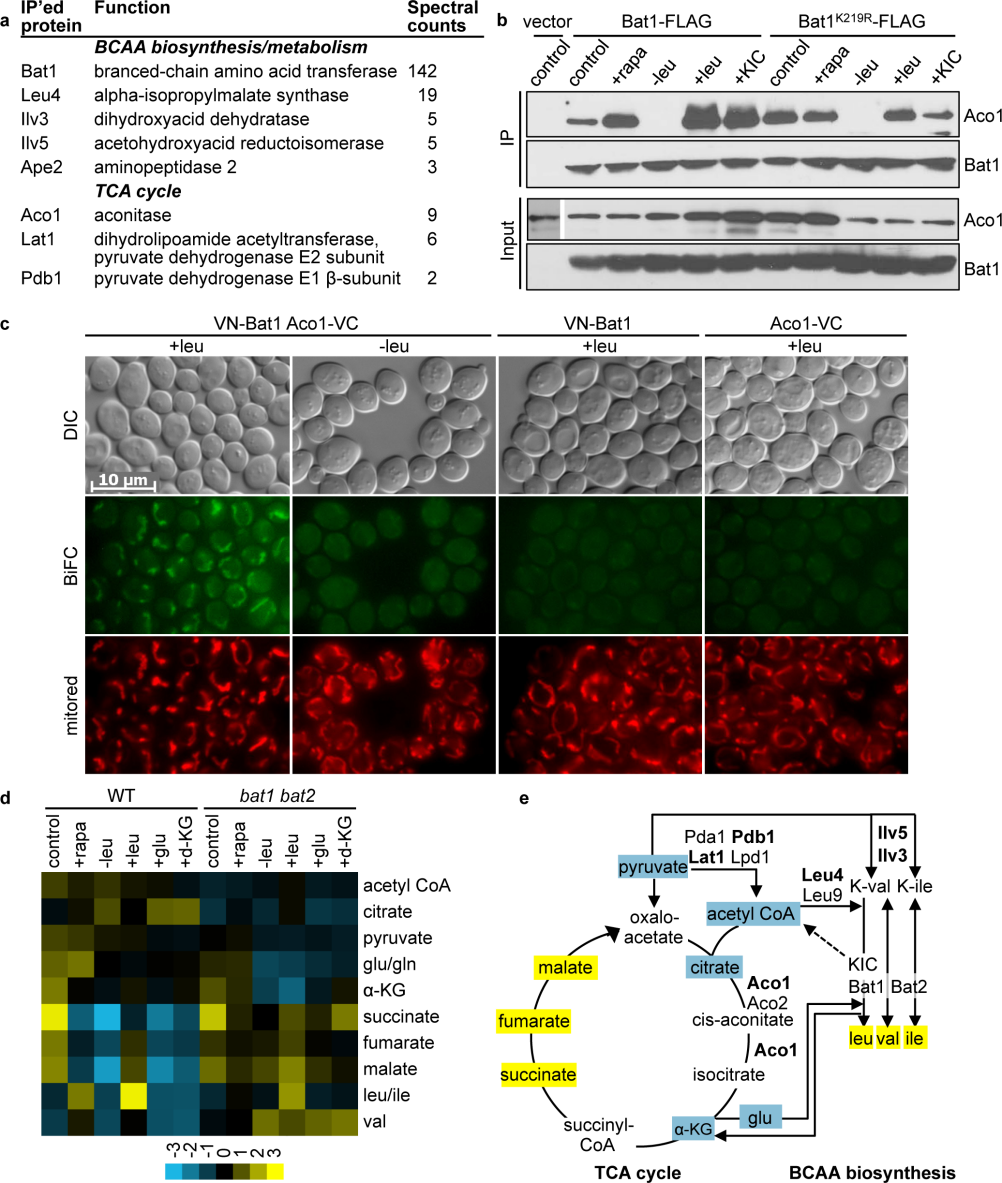
Bat1 interacts with TCA-cycle enzymes, and *bat1 bat2* mutation affects TCA-cycle metabolite levels. (**a**) The spectral counts of peptides detected by mass spectrometry for specific proteins immunoprecipitating with Bat1-FLAG are shown. (**b**) Leucine and KIC addition to leucine-starved cells fosters interaction between Bat1 and Aco1. Cells stably expressing *ACO1*-GFP and either *BAT1*-FLAG, *BAT1* ^*K219R*^-FLAG, or empty vector, were cultured in SC-ura, and harvested after the following treatments: no treatment (control), incubation with 200 nM rapamycin for 30 min (+rapa), 2 hr leucine starvation (-leu), or readdition of leucine (+leu) or KIC (+KIC) for 1 hr following 2 hr leucine starvation. Cell lysates were prepared and subjected to FLAG immunoprecipitation, followed by immunoblotting using anti-FLAG (for Bat1 and Bat1^K219R^) and anti-GFP (for Aco1) antibodies. (**c**) Bimolecular fluorescence complementation analysis of diploid cells expressing Bat1 tagged N-terminally with the N-terminal portion of Venus and under the control of the *CET1* promoter (VN-Bat1), and/or Aco1 tagged C-terminally with the C-terminal portion of Venus (Aco1-VC). Cells were incubated in either SC (+leu) or starved for leucine in SC-leu for 2 h (-leu) and visualized microscopically following treatment with MitoTracker Red CMXRos (mitored) to visualize the mitochondria. Results are representative of experiments from two independent sets of diploid strains, performed in duplicate. (**d**) Hierarchically clustered heat map of metabolite profiles of WT or *bat1 bat2* strains grown in SD supplemented with ile, leu, val and gln and treated as described in (**b**), as well as leucine, glutamine or d-KG readdition for 1 hr following 2 hr leucine starvation are shown. Hierarchical clustering was performed using Cluster 3.0 and data was visualized using scaled TreeMaps. The data are an average of 2 (acetyl CoA) or 3 (all others) independent samples. (**e**) Schematic depicting interrelationship between BCAA biosynthesis and the TCA-cycle, highlighting Bat1-interacting proteins (bold text) and metabolites altered between the WT and *bat1 bat2* mutant (coloured yellow or blue for higher or lower levels, respectively, in the *bat1 bat2* mutant compared with the WT). The valine and isoleucine α-ketoacids are annotated as K-val and K-ile. KIC conversion to acetyl CoA, depicted by a dotted line, has been shown to occur in mammals but has not been confirmed in *S*. *cerevisiae*.

We next tested the model that Bat1 plays a structural, non-enzymatic role mediated via protein-protein interactions in controlling TORC1. We employed a proteomics approach to identify Bat1-interacting proteins. To this end, Bat1-FLAG was expressed in the *bat1* mutant strain and immunoprecipitated, and Bat1-coimmunoprecipitated proteins were identified by mass-spectrometry. Specific proteins identified in the Bat1-FLAG immunoprecipitates (which were absent in control immunoprecipitates from FLAG-untagged cells) were, like Bat1, mitochondrial proteins. Interestingly, these Bat1-interacting proteins included BCAA biosynthetic and metabolic enzymes (Leu4, Ilv5, Ilv3, Ape2) and TCA-cycle enzymes including pyruvate dehydrogenase subunits (Lat1 and Pdb1) and aconitase (Aco1) (**Fig 5A**).

Our proteomic results support the intriguing model that Bat1 may comprise a central component of a metabolon linking BCAA biosynthesis to energy metabolism via the TCA-cycle. To test this model, we sought to validate the Bat1-FLAG interactions using GFP-tagged TCA-cycle pyruvate dehydrogenase and aconitase alleles. First, we confirmed that Lat1-GFP, Pdb1-GFP, and Aco1-GFP were correctly localized to the mitochondria, and had wild type function with respect to growth on respiratory carbon sources (**S2A and S2B Fig**). Because we observed a low signal for Lat1-GFP by Western blot analysis, only Aco1-GFP and Pdb1-GFP interactions were tested (**S2C Fig**). We were unable to demonstrate binding between Bat1-FLAG and Pdb1-GFP; thus this interaction may be weak, consistent with a low number of spectral reads identified by mass spectrometry (**Fig 5A**). Remarkably, Aco1-GFP readily coimmunoprecipitated with Bat1-FLAG under conditions that promote robust TORC1 activity (leucine or KIC addition), while the interaction was disrupted by leucine starvation (**Fig 5B**). The Bat1-Aco1 interaction was not perturbed by rapamycin treatment, consistent with leucine or KIC triggering this interaction upstream of TORC1. Furthermore, Bat1^K219R^-FLAG retained the ability to interact with Aco1-GFP, indicating that the interaction does not depend on pyridoxal binding or catalysis but does depend on the presence of substrate (leucine or KIC) binding (**Fig 5B**).

We also employed a bimolecular fluorescence complementation (BiFC) approach [42, 43] to test if Bat1 (fused at the N-terminus with the N-terminal-half-Venus; VC-Bat1 in *MAT* **a**) and Aco1 (fused at the C-terminus with the C-terminal-half-Venus; Aco1-VC in *MAT*α) interact with each other. Consistent with Bat1 and Aco1 interacting or occurring in close proximity to each other (within a ~7 nM distance), we observed a robust fluorescent signal that co-localized with the mitochondria when diploid cells expressing both VC-Bat1 and Aco1-VN were grown in leucine-replete media (**Fig 5C**). The signal was noticeably brighter than when control cells expressing either Aco1-VC or VC-Bat1 individually were visualized under identical conditions (**Fig 5C**). Moreover, the signal was reduced following two hours of leucine starvation, further supporting that the Bat1-Aco1 interaction is dependent on leucine (**Fig 5C**).

To determine whether Bat1 interaction with TCA-cycle enzymes is physiologically relevant, we analyzed the levels of TCA-cycle organic acid, amino acid, and acetyl CoA metabolites in the WT and *bat1 bat2* strains. Samples were prepared from control, rapamycin-treated, leucine-starved, and leucine-, KIC-, glutamate- or d-KG-addition to leucine-starved cells. We found that in general, the levels of most amino acids detected were higher in the WT compared with *bat1 bat2* mutant cells in most conditions examined, in particular upon rapamycin treatment, which blocks translation (**Fig 5D, S4 Table**). Because *bat1 bat2* mutation confers valine, leucine, and isoleucine auxotrophy, these amino acids were supplemented in the growth media; however, they are not metabolized in the *bat1 bat2* strain and thus their levels were increased in this mutant compared to the WT under most conditions tested. Interestingly, levels of acetyl CoA and the organic acids pyruvate and citrate were higher in the WT and lower in the *bat1 bat2* mutant. In contrast, succinate, fumarate, and malate were lower in the WT and higher in the *bat1 bat2* mutant. These results are consistent with a model in which the lack of BCAT results in a block in TCA-cycle flow at the entry point of the pyruvate dehydrogenase product acetyl CoA and near the point of Aco1 action. In accord with this interpretation, leucine starvation resulted in acetyl CoA and citrate accumulation concomitant with decreased levels of all of the other TCA-cycle intermediates in the WT strain. Therefore, BCATs are required to maintain metabolite homeostasis and TCA-cycle flux; likely, at least in part through their BCAA biosynthetic role, and potentially via interactions that affect activity of TCA-cycle enzymes, such as Aco1.

### Disruption of TCA-cycle flux perturbs TORC1 signaling

**Fig 6:**
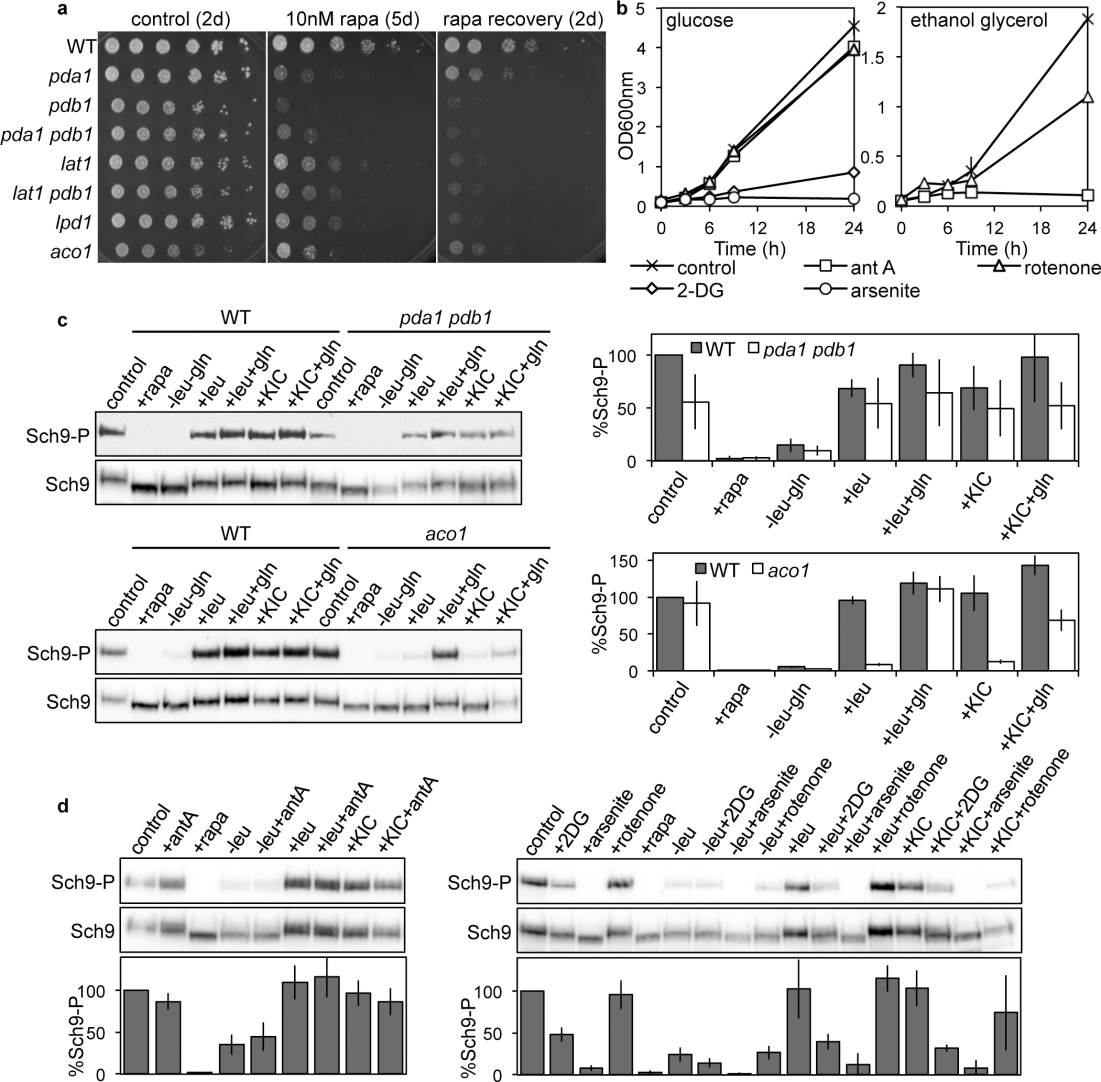
Glycolysis and TCA-cycle defects perturb TORC1 signaling. **(a)** Rapamycin sensitivity (10 nM) and recovery (following 6 h incubation) were compared for the WT, and pyruvate dehydrogenase subunit (*pda1*, *pdb1*, *lat1*) and aconitase (*aco1*) mutants grown in SC-ura, as described in **Fig 1C**. SC-ura +/- rapamycin plates were photographed after incubation for 2 or 5 days as designated. **(b)** Growth rate of the WT strain in (Glucose) or SEG (Ethanol Glycerol) media supplemented with ile, val, leu and gln, in addition to 2-deoxyglucose (2DG, 10 mM), sodium (meta)arsenite (arsenite, 5 mM), antimycin A (ant A, 50 μM) or rotenone (100 μM), as indicated. (**c**) Mutation of pyruvate dehydrogenase subunits (*pda1 pdb1*) and aconitase (*aco1*) impaired TORC1 kinase activity. Strains were cultured in SD+ile+val+leu+gln, and protein extracts were prepared following no treatment, rapamycin incubation (200 nM, 30 min), 2 hr leucine starvation without glutamine, or readdition of leucine or KIC +/- glutamine for 1 hr following 2 hr leucine starvation. (**d**) Inhibition of glycolysis and the TCA-cycle (2DG and sodium (meta)arsenite treatments, respectively), inhibit TORC1 kinase activity. Sch9 phosphorylation was monitored following treatment of WT cells with designated inhibitors for 30 min, or for 30 min prior to leucine or KIC addition following leucine starvation. (**c**, **d)** Sch9 phosphorylation was determined and quantified as described in **Fig 1D**. All experiments were performed in triplicate.

We next tested if inhibition of TCA-cycle flux perturbs TORC1 signaling. First, we determined the effects of disruption of genes encoding Bat1-interacting pyruvate dehydrogenase subunits (*pda1*, *pdb1*, *lat1*, *lpd1*) or aconitase (*aco1*) on TORC1-related phenotypes. Compared with the WT, all mutants were rapamycin hypersensitive and had impaired recovery from rapamycin-induced growth arrest (**Fig 6A**). Furthermore, the *pda1 pdb1* mutant also had reduced TORC1 activity under all conditions tested; for example, Sch9 phosphorylation levels were reduced to 50% of WT in cells grown under control conditions (**Fig 6C**). The *aco1* mutant also had substantially reduced TORC1 activity (15–20% of WT levels) following leucine or KIC addition to leucine-starved cells. Mutation of *ACO1* renders cells auxotrophic for glutamate [44, 45]; thus, we reasoned that glutamine levels should also be reduced in these mutants. Accordingly, simultaneous addition of glutamine with leucine or KIC resulted in TORC1 activation to a level comparable to the WT strain (**Fig 6C**).

We also assessed the effect of inhibitors of glycolysis (2DG, which reduces pyruvate feeding into the TCA-cycle) and the TCA-cycle (sodium (meta)arsenite) on TORC1 activity. Both 2DG and sodium (meta)arsenite inhibited growth at the concentrations tested (**Fig 6B**). TORC1 activity was then assessed for WT cells in which inhibitors were added during control-growth conditions and during leucine starvation 30 min prior to addition of leucine or KIC. Sodium (meta)arsenite and 2DG markedly reduced levels of TORC1 activity under all conditions tested (**Fig 6D**). To determine if TCA-cycle flux controls TORC1 via increased flux through the electron transport chain, we also assessed the effect of electron transport chain inhibitors (rotenone, antimycin A) on TORC1 activity. Antimycin A only inhibited growth in non-fermentable carbon sources (ethanol glycerol medium), while rotenone had little effect on growth (**Fig 6B**), consistent with a rotenone-insensitive NADH:Q6 oxidoreductase identified previously in yeast [46]. In accord with these results, neither rotenone nor antimycin A perturbed TORC1 activity (**Fig 6D**) indicating that flux through the TCA-cycle, but not the respiratory chain, controls TORC1 signaling under our experimental conditions.

### A role for ATP levels and Snf1 in BCAT control of TORC1

We considered three hypotheses to explain the mechanism by which the integration between BCATs and TCA-cycle fluxes could influence TORC1 signaling. First, we reasoned that acetyl CoA could affect TORC1 activity by fueling the TCA-cycle. In mammalian cells, KIC can be converted to acetyl CoA via branched-chain ketoacid dehydrogenase complex (BCKDC) activity. However, no BCKDC enzymes or activity have been identified in yeast. Although acetyl CoA levels decreased upon leucine starvation (**Fig 5D**), addition of acetate, which increases cytosolic acetyl CoA levels [47], failed to stimulate TORC1 activity in leucine-starved WT cells (**S1C Fig**).

Second, TCA-cycle fluxes may signal to TORC1 by affecting glutamine levels, which in turn activates TORC1 independently of the EGOC [14]. Glutamine addition greatly but not completely overcame the TORC1 signaling defects in the *aco1* mutant, and also increased TORC1 activity when added with leucine or KIC to WT cells following leucine starvation (**Fig 6C**). However, glutamine did not elevate TORC1 activity in either the *pda1 pdb1* (**Fig 6C**) or *bat1 bat2* (**Fig 1F**) mutants when supplemented in the same conditions. These results suggest that while glutamine provides a contribution, there must be additional inputs.

**Fig 7:**
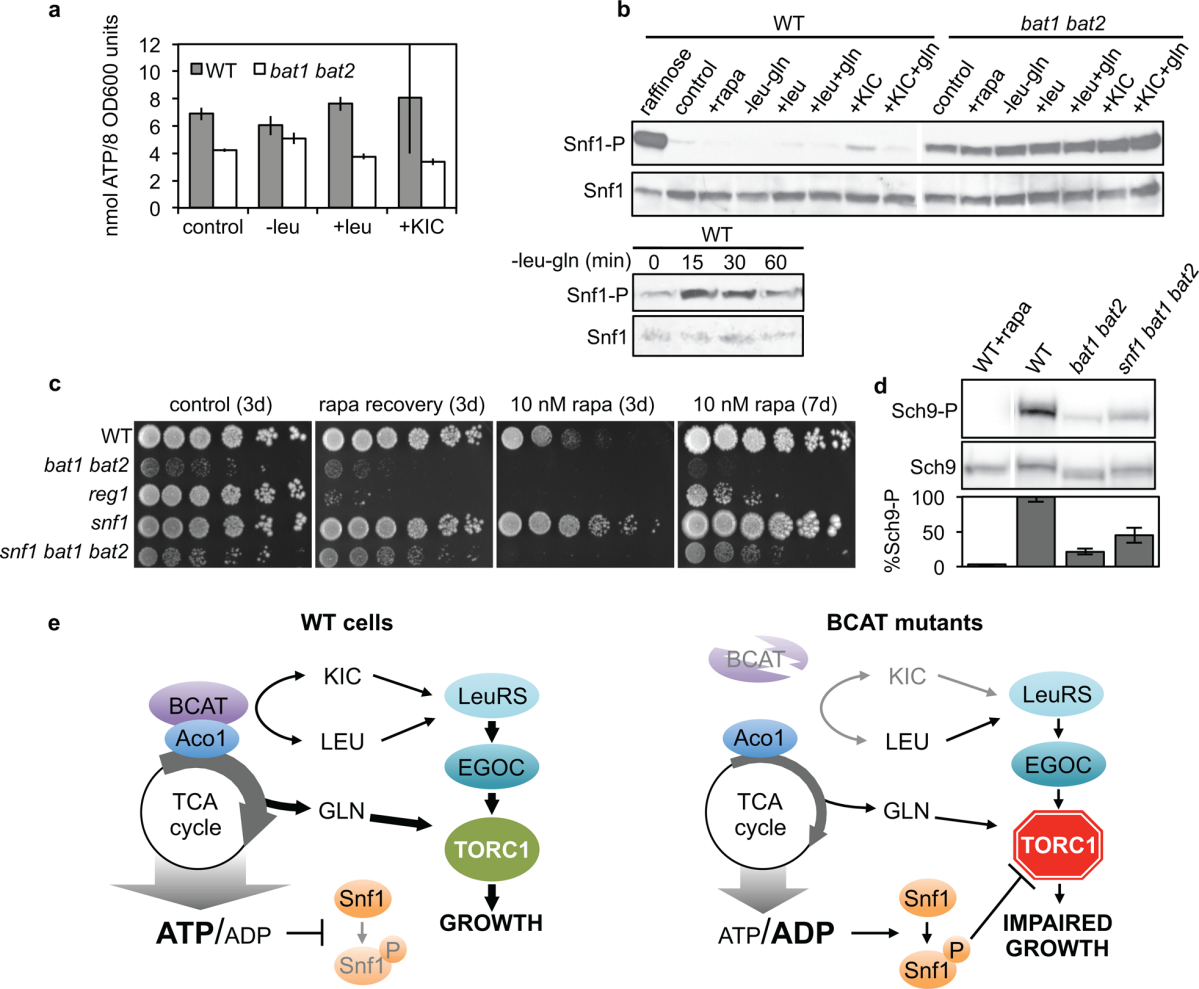
BCAT mutation reduces ATP levels and elevates Snf1 phosphorylation. (**a**) Relative ATP levels from duplicate extracts of WT and *bat1 bat2* cells cultured in SD+ile+val+leu+gln media (control) and leucine-starvation and leucine and KIC readdition conditions described in **Fig 1B**. (**b**) Phosphorylation of Snf1 Thr210, assessed by immunoblotting with a phosphospecific antibody against mammalian phospho-Thr172-AMPK (Snf1-P panel). Strains were cultured as described in **Fig 6C**, and following 1 hr incubation in S-Raffinose+ile+val+leu as a positive control for Snf1 phosphorylation, as indicated (upper panel) or following starvation for leucine for the times indicated (lower panel). Membranes were stripped and reblotted with anti-Snf1 antibody (Snf1 panel) to ensure uniform loading. The experiment was performed in triplicate. (**c**) Rapamycin sensitivity and recovery of strains plated to YPD (control, rapa recovery) or YPD supplemented with 10 nM rapamycin, was determined as described in **Fig 1C**. (**d**) Sch9 phosphorylation of strains cultured in SD+ile+val+leu+gln, with rapamycin addition where indicated, was determined as described in **Fig 1D.** (**e**) Model for BCAT control of TORC1 activity. BCATs interconvert leucine and KIC, which are both signaled to TORC1 via LeuRS and EGOC. BCATs also promote robust TORC1 activity by maintaining TCA-cycle flux through their biosynthetic role and via interaction with TCA-cycle enzyme Aco1, elevating ATP and glutamine levels, which promote TORC1 activity via EGOC-independent routes. Reduced TCA-cycle flux in BCAT mutants results in: 1) reduced glutamine levels, decreasing TORC1 activity, and 2) an elevated ADP:ATP ratio, which activates Snf1 through phosphorylation, and activated Snf1-P mediates further TORC1 inhibition.

Third, reduced TCA-cycle flux should result in increased AMP:ATP or ADP:ATP ratios, which in turn trigger TORC1 inhibition by a cascade involving activation of the AMP-activated kinase (AMPK) Snf1 [21] via Thr210-phosphorylation. Indeed, we found that *bat1 bat2* mutants growing in leucine-replete conditions had reduced ATP levels compared with the WT (**Fig 7A**). We tested if the observed perturbations in ATP levels were sufficient to foster activation of Snf1 phosphorylation at residue Thr210 by employing a phospho-specific antibody against mammalian AMPK Thr172 that effectively cross-reacts with phospho-Snf1 Thr210 [48]. Interestingly, in general we observed markedly elevated Snf1 phosphorylation in *bat1 bat2* cells compared with the WT under all conditions tested in glucose-containing media (**Fig 7B** upper panel) and a moderate elevation of Snf1 phosphorylation in the WT strain upon leucine starvation (**Fig 7B** lower panel). Consistent with a TORC1-inhibitory role of Snf1, mutation of *SNF1* led to rapamycin resistance compared with the WT (**Fig 7C** panel 3). Conversely, mutation of the Glc7 protein phosphatase regulator *REG1*, which promotes Snf1 dephosphorylation [49], resulted in rapamycin hypersensitivity (**Fig 7C**), confirming earlier reports [50, 51]. Moreover, *snf1* disruption partially suppressed the rapamycin sensitivity and recovery (**Fig 7C**) and TORC1 activity defects (**Fig 7D**) of the *bat1 bat2* mutant strain. These results support a model whereby BCATs control TCA-cycle flux and thereby high ATP levels to promote robust TORC1 activity. BCAT disruption leads to an elevated ADP:ATP ratio, which in turn results in Snf1 activation and TORC1 inhibition (**Fig 7E**).

## Discussion

The amino acid leucine is a potent inducer of both yeast and mammalian TORC1 activity, but the mechanisms by which regulation occurs are not fully understood. Here, we demonstrate that, similar to leucine, KIC is able to activate TORC1 via an EGOC-dependent mechanism. In addition leucine and KIC, in combination with BCAT, play novel EGOC-independent roles in activating TORC1 signaling. Consistent with an EGOC-dependent role, we provide evidence that KIC is sensed by the leucyl-tRNA synthetase (LeuRS) in a mechanism analogous to leucine and leucinol sensing (**Fig 2C**), reported to act upstream of EGOC or Rag GTPases to signal leucine availability to TORC1 [8, 9].

Our demonstration of KIC stimulation of yeast TORC1 activity has precedent in controlling mTORC1. KIC, but not other BC-α-ketoacids, activates mTORC1 similar to leucine in different mammalian models [30–33] and, like leucine, KIC partially rescues development in a zebrafish model of Cornelia de Lange syndrome in a TORC1-dependent manner [52]. Compared with leucine, KIC stimulates mTORC1 activity more efficiently in rat skeletal muscle than in liver, which contains considerably lower mammalian BCAT activity levels. These results were interpreted as consistent with a requirement of BCAT-mediated conversion of KIC to leucine for promoting mTORC1 activity [31]. In contrast, our experiments show KIC and leucine activate yeast TORC1 via both BCAT-dependent and independent mechanisms, and it would be of interest to investigate if a similar BCAT-independent TORC1 activation mechanism occurs in mammals.

We present evidence for both enzymatic and structural roles for BCATs in controlling TORC1 activity. Mitochondrial Bat1 is preferentially expressed during the logarithmic phase of growth when energy is produced by glycolysis while cytosolic Bat2 is expressed during stationary growth phase when energy is obtained via respiration [41, 53]. Given the differential Bat1 and Bat2 subcellular localization and temporal expression, we propose BCAT enzymatic activity could control TORC1 signaling by perturbing the levels of BCAA and central metabolites, glutamate and α-ketoglutarate, in the cytosol and the mitochondria during specific growth phases. Our results suggest that the TORC1-relevant structural role of Bat1, but not of Bat2, involves a direct physical interaction with other BCAA-biosynthetic enzymes (Ilv5, Ilv3, Leu4) as well as the TCA-cycle PDH and aconitase Aco1. Such a multiprotein metabolon may provide an assembly line to couple leucine metabolism and glycolysis products to TCA-cycle fluxes, energy production, and TORC1 signaling (**Fig 5E**). Strikingly, we find the interaction of Bat1 and Aco1 is disrupted by leucine starvation, and reestablished following either leucine or KIC readdition (conditions that also support TORC1 activity). Although the physiological effects of Bat1 interaction on Aco1 activity remain to be determined, we show that BCAT mutants have perturbed TCA-cycle intermediate levels consistent with a block in the TCA-cycle pathway at the step of pyruvate and acetyl-CoA incorporation. Taken together, we propose that BCATs may affect TORC1 activity by controlling TCA-cycle flux via physical interactions with Aco1 and possibly PDH, and by contributing metabolites to fuel the cycle.

Multiprotein complexes consisting of mitochondrial BCAT (BCATm) and TCA-relevant enzymes, as reported here for yeast Bat1, have also been identified in mammals [38, 54]. BCATm interacts with pyruvate carboxylase and the branched-chain α-keto acid dehydrogenase enzyme complex (BCKDC), which converts BCAA-derived α-ketoacids to TCA-cycle intermediates including branched-chain acyl-CoAs, acetyl-CoA, or succinyl-CoA [38, 54]. Although yeast lacks BCKDC and BC-α-ketoacid degradation instead occurs via the Ehrlich pathway [37] (in a reaction where Bat2, as opposed to Bat1, is more predominant [55]), the BCKDC-related PDH complex, which our results suggest interacts with Bat1 (**Fig 5**), catalyzes an analogous set of reactions as the BCKDC, except with pyruvate instead of α-keto acids as substrates [56]. BCATm-pyridoxal-5-phosphate (reduced) interacts with BDKDC, while BCATm-pyridoxamine 5-phosphate (oxidized by the transamination reaction, which requires substrate presence) binds and activates Gdh1, thereby coupling the BCAA-mediated amination of α-ketoglutarate to form glutamate with the regeneration of BCATm-pyridoxal-5-phosphate and α-ketoglutarate [38, 39, 54], and this reaction which may also contribute to mTORC1 activation [29]. Interestingly, while pyridoxal/pyridoxamine-phosphate binding defines BCATm interactions with BCKDC and Gdh1 [41, 57–59], Aco1-Bat1 interaction is not governed by pyridoxal phosphate-binding and our results show that this interaction is triggered by leucine and KIC.

We propose two mechanisms via which Bat1 stimulation of TCA-cycle flux controls TORC1 activity independently of the EGOC (**Fig 7E**). First, increasing α-ketoglutarate levels may sustain glutamine production, which in turn activates TORC1 activity in an EGOC-independent fashion [14]. In contrast to proliferating mammalian cells wherein glutaminolysis predominates over glutamine and glutamate synthesis to produce α-ketoglutarate that sustains TCA-cycle flux [60], the equilibrium in glucose-grown yeast instead favors glutamate and glutamine synthesis from α-ketoglutarate for anabolic reactions [61, 62]. Furthermore, while glutaminolysis drives mTORC1 activity [29], glutamine synthesis and accumulation activate yeast TORC1 [14]. However, whereas glutamine addition restored TORC1 activity to an *aco1* mutant (**Fig 6C**), it failed to stimulate TORC1 activity in the *bat1 bat2* mutant (**Fig 1F**), suggesting another TORC1-inhibitory mechanism occurs in this strain. This BCAT-dependent mechanism likely involves a role for maintenance of ATP levels to sustain TORC1 activity. Previously, mTORC1 was proposed to act directly as an ATP sensor by virtue of its unusually high K_m_ for ATP (>1 mM) [63] compared with most kinases (10–20 μm) [64], although others have argued that the intracellular ATP concentration is normally higher than 1 mM, and a substantial change in ATP levels would be required to alter mTORC1 activity [65]. A high ratio of AMP:ATP in mammals or ADP:ATP in yeast, reflecting low energy production, activates the conserved AMPK/Snf1 by phosphorylation to induce pathways for energy production and downregulate pathways for energy consumption [66–68]. In mammals, AMPK is thought to be a more sensitive ATP sensor than mTORC1 because the intracellular AMP concentration is significantly lower than ATP, and thus, small changes to ATP levels could substantially affect the ATP:AMP ratio [69]. We find that BCAT disruption led to both reduced ATP levels, and increased Snf1 phosphorylation. Substantial research demonstrates that AMPK activation inhibits mTORC1 by phosphorylation of the mTORC1 subunit RAPTOR and the negative mTORC1 regulator tuberous sclerosis complex 2 [70–72]. Recent research also supports a role for Snf1 involvement in yeast TORC1 inhibition [20, 21]. Upon glucose starvation, Snf1 phosphorylates and activates PAS kinase signaling, which in turn phosphorylates Pbp1 and this event correlates with TORC1 inhibition [21]. Alternatively, Snf1 could control TORC1 by phosphorylation of Kog1 and Tco89, both of which were recently identified as Snf1 targets [73]. Our results support the model that the AMPK/Snf1 cascade inhibits TORC1 in yeast BCAT mutants. This mechanism for TORC1 inhibition could operate at the diauxic shift and during stationary growth phase when glucose is exhausted and Bat1 is poorly expressed [53], likely resulting in AMPK/Snf1 activation, which in turn could fine-tune gene expression necessary for energy saving (notably, downregulation of TORC1-controlled ribosome biogenesis) while promoting respiratory capacity (induction of genes for utilization of alternative carbon sources and aerobic growth [74]. However, further experimentation will be required to test this model.

As in yeast, both the cytoplasmic BCATc and mitochondrial BCATm isoenzymes are present in mammals, differentially expressed, and control different aspects of leucine metabolism. BCATc expression is restricted to selected neurons but induced in regulatory T-cells, skin grafts, proliferating embryonic and cancer cells [75–78] and may be a prognostic marker for aggressive glioblastomas carrying WT IDH [79]. Most notably, BCAT overexpression has been associated with brain, urothelial, bladder, and breast cancers, for which treatment or clinical trials with the rapamycin analog everolimus have been approved or are underway, respectively, highlighting the coincidence of BCAT and TORC1 defects in human disease [79–81]. Interestingly, mutation of BCATc increased glycolytic metabolism, leucine levels, and mTORC1 activity in activated T-cells [82]. In contrast to BCATc, metabolon-forming BCATm is expressed in most body tissues with the exception of the liver and coincident with BCKDC presence [76]. Furthermore, evidence exists that α-ketoglutarate generated from glutaminolysis, governed by the interaction between BCATm and leucine-allosterically activated Gdh1 [38, 39], stimulates mTORC1, thereby also integrating both glutamine and leucine signals [29]. Therefore, BCATc and BCATm may differently contribute to control mTORC1. Given the ubiquitous distribution of TORC1 and BCATs in yeast and mammals and the conserved role of BCATm in forming multicomplex metabolons connecting BCAA, glycolysis, and TCA-cycle metabolism, we predict that our findings in yeast will have implications for mTORC1 signaling in human health and disease.

## Materials and Methods

### Strains, media and growth conditions

Media consisted of Yeast Extract Peptone Dextrose (YPD), Synthetic Complete (SC), Synthetic Dextrose (SD), Synthetic Ethanol Glycerol (SEG) or synthetic medium with amino acids and supplements omitted or added to complement auxotrophies or select for plasmid maintenance [83, 84]. Synthetic (S)-Raffinose contained 20 g/L raffinose as the sole carbon source. When required, media was supplemented with rapamycin (LC Laboratories), 100 μg/ml nourseothricin (ClonNAT, Werner BioAgents), 200 μg/ml G418 (AG Scientific), 200 μg/ml hygromycin (Calbiochem), and 2 mM α-ketoisocaproate, 2 mM dimethyl α-ketoglutarate (d-KG), 2 mM isovaleraldehyde, 2 mM isoamyl alcohol, 10 μM 1,3-dihydro-1-hydroxy-2,1-benzoxaborole (DHBB), 50 μM antimycin A, 10 mM 2-deoxyglucose (2DG), 5 mM sodium (meta)arsenite, 100 μM rotenone, or potassium acetate (Sigma). All cultures were incubated at 30°C.

Strains used in this study are derived from BY4742 or BY4741 [85] and are listed in **S1 Table**. To construct strains employed in the BiFC assay, the N-terminal of Bat1 was fused with the N-terminal-half-Venus (VN) (under the control of the mid-strength *CET1* promoter) in BY4741 (*MAT* **a**), and the C-terminal of Aco1 was fused with the C-terminal-half-Venus in BY4742 (*MAT*α). Following PCR-confirmation, strains were crossed to generate a diploid co-expressing both fusion proteins, or control diploid strains expressing either fusion individually.

### Plasmid construction

Plasmids and oligonucleotides used in this study are listed in **S2** and **S3 Tables**, respectively. To construct pPC02, the *BAT1* gene was PCR-amplified from BY4742 genomic DNA using oligonucleotides that contained XbaI and BamHI sites (MPDC01 and MPDC02) and the XbaI/BamHI-digested PCR product was cloned into the XbaI/BamHI-digested vector, p416ADH. In an analogous fashion, *BAT2*, amplified using oligonucleotides MPDC13 and MPDC14, was cloned in p416ADH to produce plasmid pJK51. Construction of the *BAT1* ^K219R^ and *BAT1* ^K219A^ alleles was performed as follows using mutagenic oligonucleotides. The 5’ and 3’ ends of *BAT1* ^K219R^ were amplified using oligonucleotide pairs MPDC01+JK46 and MPDC02+JK44, and of *BAT1* ^K219A^ using MPDC01+JK47, MPDC02+JK45, respectively. Based on the complementarity of oligonucleotides JK44 with JK46, and JK45 with JK47, purified PCR products were used as templates in an overlap PCR reaction (oligonucleotides MPDC01+MPDC02). The final overlap *BAT1* ^K219R^ and *BAT1* ^K219A^-containing PCR products were XbaI/BamHI-digested and cloned into XbaI/BamHI-digested p416ADH to yield pJK12 and pJK15, respectively. Similarly, the 5’ and 3’ ends of the *BAT2* ^*K202A*^ and *BAT2* ^*K202R*^ alleles were amplified using oligonucleotide pairs MPDC13+JK372 and MPDC14+JK371, and MPDC13+JK370 and MPDC14+JK369, respectively. Fusion products amplified using oligonucleotides MPDC13+MPDC14 were cloned into p416ADH to create pJK48 (*BAT2* ^*K202A*^) and pJK50 *BAT2* ^*K202R*^. To construct the FLAG-tagged *BAT1* and *BAT1* ^K219R^ plasmids pJK47 and pJK59, *BAT1-*FLAG and *BAT1* ^K219R^-FLAG were PCR-amplified from genomic or pJK12 DNA using oligonucleotides MPDC01+JK376, and XbaI/BamHI-digested products were ligated into XbaI/BamHI-digested p416ADH. All plasmids were confirmed by restriction digest analysis and sequencing of the insert.

### Microscopy

BiFC Venus signal images were taken using a standard fluorescein isothiocyanate filter with identical exposure settings for each strain and condition, and processed using identical levels of image contrast using ImageJ software. To visualize mitochondria, 100 nM MitoTracker Red CMXRos (Molecular Probes) was added to cells for the final hour of incubation. Twice-washed cells were imaged using a Zeiss Axioskop 2 Plus microscope and AxioVision 4.6 image acquisition software.

### Protein extraction and western blot analysis

Protein extract preparation, Sch9 phosphorylation, and FLAG coimmunoprecipitation assays were performed as described previously [86]. Western blot analysis employed Sch9 and phospho-Thr737-Sch9 [86], Bat1 and Bat2 [41], FLAG (Sigma), GFP (Roche), Snf1 (Santa Cruz Biotechnology, Inc.), and phospho-Thr172-AMPKα (Cell Signaling Technology) antibodies.

### Protein mass spectrometry

Protein mass spectrometry was performed by the Duke University Medical Center Proteomics and Metabolomics Core Facility, as follows. The eluents from FLAG-affinity immunoprecipitates were subjected to a 3 min SDS-PAGE separation on an Invitrogen NuPAGE 4–12% gel for desalting purposes and stained with colloidal Coomassie stain. Gel bands were excised and subjected to in-gel reduction with 5 mM dithiothreitol and alkylation with 10 mM iodoacetamide. Trypsin digestion (sequencing grade, Promega Corp) was allowed to proceed overnight at 37°C. Following peptide extraction, peptides were vacuum centrifuged to dryness and resuspended in 12 μl of 1% TFA/2% acetonitrile. LC/MS/MS was performed on 2 μl of each sample using a nanoAcquity UPLC system (Waters Corp) coupled to a Thermo QExactive Plus high-resolution accurate mass tandem mass spectrometer (Thermo) via a nanoelectrospray ionization source. Briefly, the sample was first trapped on a Symmetry C18 300 mm × 180 mm trapping column (5 μl/min at 99.9/0.1% v/v water/acetonitrile), after which the analytical separation was performed using a 1.7 μm Acquity BEH130 C18 75 mm × 250 mm column (Waters Corp) using a 90 min gradient of 5 to 40% acetonitrile with 0.1% formic acid at a flow rate of 400 nL/min with a column temperature of 55°C. Data collection on the QExactive Plus mass spectrometer was performed in a data-dependent acquisition (DDA) mode of acquisition with a r = 70,000 (at m/z 200) full MS scan from m/z 375–1600 with a target AGC value of 1e6 ions followed by 10 MS/MS scans at r-17,500 (at m/z 200) at a target AGC value of 5e4 ions. A 20s dynamic exclusion was employed to increase depth of coverage.

Raw LC-MS/MS data files were processed in Proteome Discoverer and then submitted to independent Mascot searches (Matrix Science) employing the SwissProt database (Yeast taxonomy) containing both forward and reverse entries of each protein. Search tolerances were 5 ppm for precursor ions and 0.02 Da for product ions using trypsin specificity with up to two missed cleavages. Carbamidomethylation (+57.0214 Da on C) was set as a fixed modification, whereas oxidation (+15.9949 Da on M) and deamidation (+0.98 Da on NQ) were dynamic modifications. All searched spectra were imported into Scaffold (v4.4, Proteome Software) and scoring thresholds were set to achieve a protein false discovery rate of 1.0% using the PeptideProphet algorithm.

### Metabolite analysis

Amino acids and organic acids were extracted from 8 OD_600nm_ units of cells grown to OD_600nm_~0.8 in SD+ile+leu+val+gln, and subjected to various treatments. Cells were filtered on 1.2 μm nitrocellulose filters (Millipore), washed twice with 5 ml sterile dH_2_0, resuspended in 1 ml ice-cold methanol, and then pellets were dried on a Speed-vac at room temperature. Pellets were resuspended in ice-cold methanol or 50% acetonitrile/0.3% formic acid for analysis of amino and organic acids, respectively. ATP and acetyl CoA were extracted from 8 OD_600nm_ units of cells as described previously [87], with modifications [88], and pellets for acetyl CoA analysis were resuspended in 50% acetonitrile/0.3% formic acid for analysis.

Metabolomic analyses were performed by the Sarah W. Stedman Nutrition and Metabolism Center, Duke University Medical Center, as follows. Amino acids, organic acids, and acetyl CoA were analyzed using stable isotope dilution techniques. Amino acid measurements were made by flow injection tandem mass spectrometry using sample preparation methods described previously [89, 90]. The data were acquired using a Waters Acquity UPLC system equipped with a TQ (triple quadrupole) detector and a data system controlled by MassLynx 4.1 operating system (Waters, Milford, MA). Organic acids were quantified using methods described previously [91] employing Trace Ultra GC coupled to ISQ MS operating under Xcalibur 2.2 (Thermo Fisher Scientific, Austin, TX). Acetyl CoA was extracted and purified as described previously [92, 93], and analyzed by flow injection analysis using positive electrospray ionization on Xevo TQ-S, triple quadrupole mass spectrometer (Waters, Milford, MA).

ATP concentration was determined using the ATP Colorimetric/Fluorometric Assay Kit (Sigma) from extracts resuspended in 100 μl ATP Extract Buffer, as recommended by the manufacturer.

## Supporting Information

S1 Fig Leucine biosynthetic intermediate accumulation and acetate addition do not affect TORC1 signaling. **(a)** Sch9 phosphorylation was determined in triplicate for strains that were cultured in SC+gln with treatments and methodology described in **Fig 1**. (**b**) A partial depiction of the leucine biosynthetic pathway is shown. **(c)** Acetate addition does not stimulate TORC1 activity of the WT following leucine starvation. Sch9 phosphorylation was determined in triplicate as in **Fig 1** for strains grown in SC-his-ura-lys+gln, rapa treatment, leucine starvation and readdition of leucine or potassium acetate (in the absence of leucine) at the concentrations indicated for 1 hr following 2 hr leucine starvation.
(TIF)

S2 Fig GFP-tagged Pdb1 Lat1 and Aco1 have WT localization and function, and are stably expressed. (**a**) Mitochondrial localization of GFP-tagged Aco1, Pdb1, and Lat1 was visualized microscopically following growth of strains in SC medium to which 100 nM MitoTracker Red CMXRos (Molecular Probes) was added for the final hour of incubation. Cells were washed twice and imaged using a Zeiss Axioskop 2 Plus microscope and AxioVision 4.6 image acquisition software. (**b**) Five-fold serially diluted cultures were plated onto SC and S-ethanol glycerol media and incubated for the times indicated. (**c**) Cell lysates from strains expressing *ACO1*-GFP, *LAT1*-GFP, *PDB1*-GFP, and/or *BAT1*-FLAG were prepared and subjected to western blot analysis using anti-FLAG (for Bat1) and anti-GFP (for Aco1, Lat1 and Pdb1) antibodies.
(TIF)

S1 Table Yeast strains used in this study. (DOCX)

S2 Table Plasmids used in this study. (DOCX)

S3 Table Oligonucleotides used in this study. (DOCX)

S4 Table Relative amounts of metabolite abundance in WT and *bat1 bat2* yeast strains. Average metabolite levels from cell extracts prepared from cultures grown in SD+ile leu val gln (T0 control), rapamycin treatment (200 nM, 30 min, +Rapa), 2 hr leucine starvation (-Leu), and addition for 1 hr following leucine starvation of leucine, glutamine, or d-KG, as indicated. Extracts were prepared in triplicate for amino acids and organic acids, and duplicate for acetyl CoA.
(DOCX)
