# Supplementary figures and images for: Branched-Chain Aminotransferases Control TORC1 Signaling in Saccharomyces cerevisiae
Source: PLoS Genet. 2015 Dec 11;11(12):e1005714. doi: 10.1371/journal.pgen.1005714 (PMC4684349; doi:10.1371/journal.pgen.1005714)

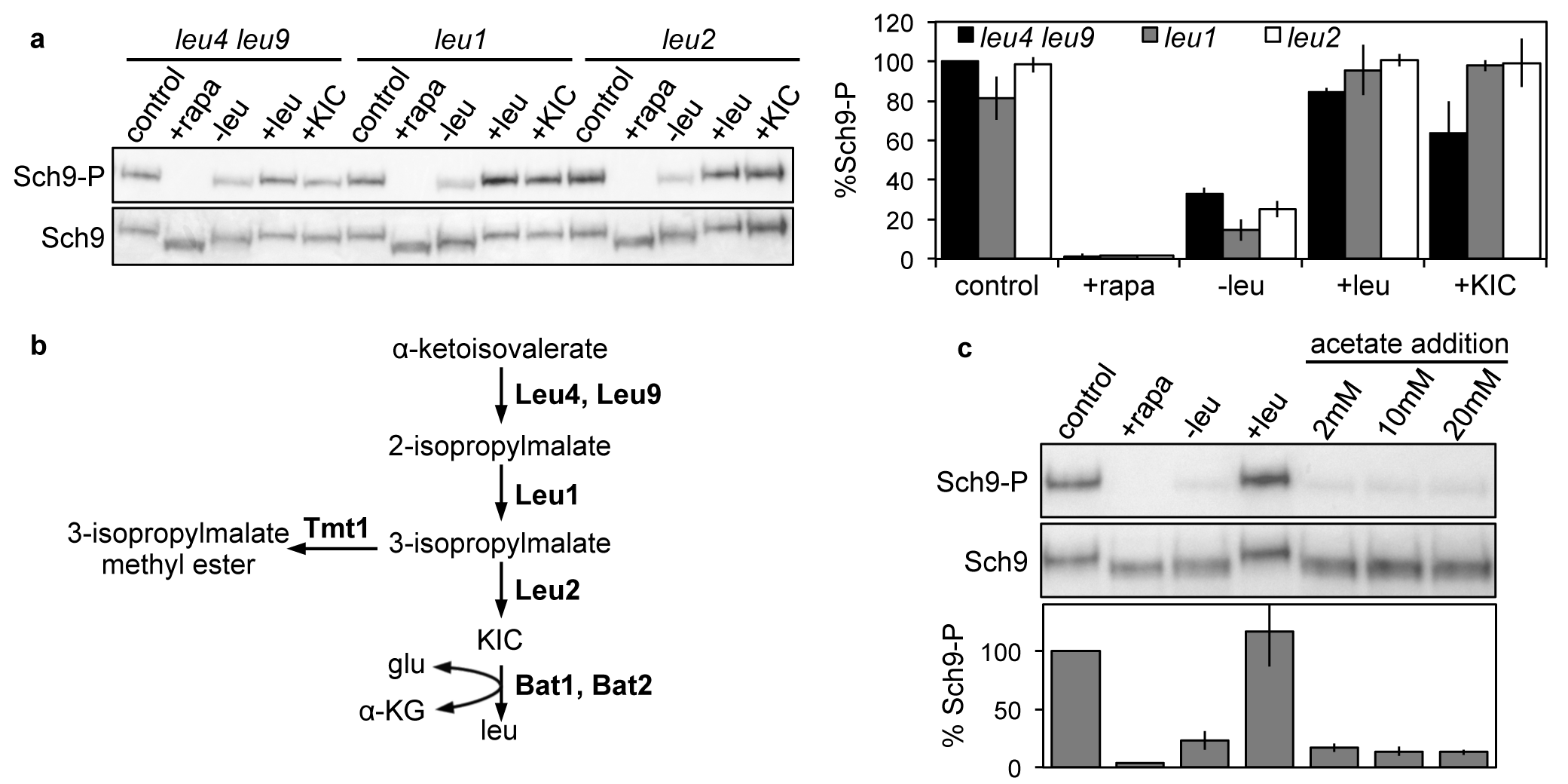

Supplement: S1 Fig — (a) Sch9 phosphorylation was determined in triplicate for strains that were cultured in SC+gln with treatments and methodology described in Fig 1. (b) A partial depiction of the leucine biosynthetic pathway is shown. (c) Acetate addition does not stimulate TORC1 activity of the WT following leucine starvation. Sch9 phosphorylation was determined in triplicate as in Fig 1 for strains grown in SC-his-ura-lys+gln, rapa treatment, leucine starvation and readdition of leucine or potassium acetate (in the absence of leucine) at the concentrations indicated for 1 hr following 2 hr leucine starvation. (TIF) [file pgen.1005714.s001.tif]

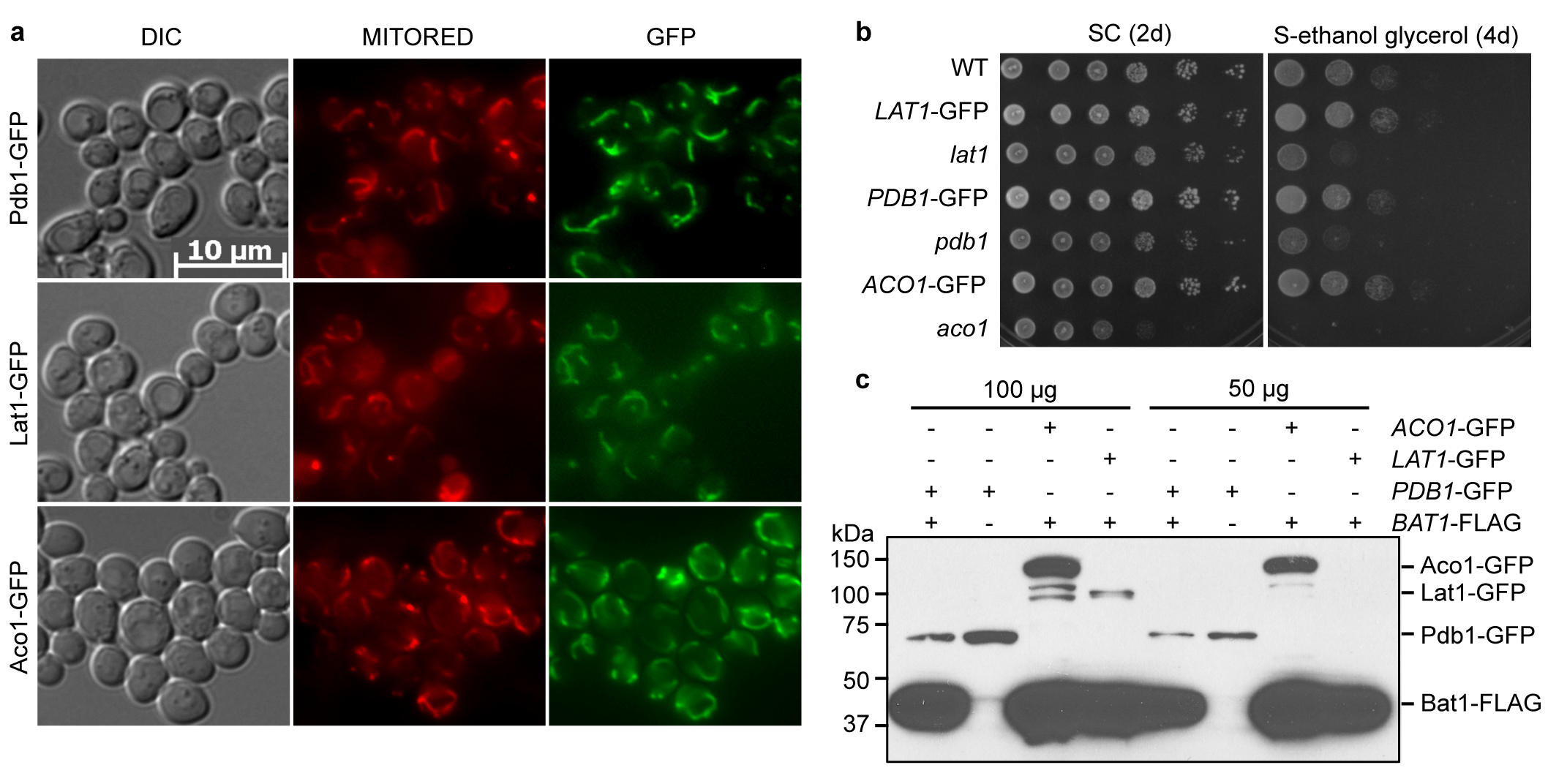

Supplement: S2 Fig — (a) Mitochondrial localization of GFP-tagged Aco1, Pdb1, and Lat1 was visualized microscopically following growth of strains in SC medium to which 100 nM MitoTracker Red CMXRos (Molecular Probes) was added for the final hour of incubation. Cells were washed twice and imaged using a Zeiss Axioskop 2 Plus microscope and AxioVision 4.6 image acquisition software. (b) Five-fold serially diluted cultures were plated onto SC and S-ethanol glycerol media and incubated for the times indicated. (c) Cell lysates from strains expressing ACO1-GFP, LAT1-GFP, PDB1-GFP, and/or BAT1-FLAG were prepared and subjected to western blot analysis using anti-FLAG (for Bat1) and anti-GFP (for Aco1, Lat1 and Pdb1) antibodies. (TIF) [file pgen.1005714.s002.tif]
