# Supplementary material for: Branched-Chain Aminotransferases Control TORC1 Signaling in Saccharomyces cerevisiae
Source: PLoS Genet. 2015 Dec 11;11(12):e1005714. doi: 10.1371/journal.pgen.1005714 (PMC4684349; doi:10.1371/journal.pgen.1005714)
Supplement: S1 Table — (DOCX) [file pgen.1005714.s003.docx]

**Table S1** Yeast strains used in this study.

| **Strain** | **Genotype^a^** | **Figure** |
| --- | --- | --- |
| BY4742 | *MAT*α *his3*Δ*1 leu2*Δ*0 lys2*Δ*0 ura3*Δ*0* |  |
| BY4741 | *MAT***a** *his3*∆*1* *leu2*∆*0* *met15*∆*0* *ura3*∆*0* |  |
| YJK3850 | pPC10 pMKV002 | 1b, d-f, 2b, c, 3b, c, 4b, 5d, 6, 7, S1 |
| YJK4221 | *thi3*∆::kanMX4 *aro10*∆::natMX4 pPC10 pMKV002 | 1c, d |
| YJK4222 | *aro10*∆::kanMX4 pPC10 pMKV002 | 1c |
| YJK4223 | *thi3*∆::kanMX4 pPC10 pMKV002 | 1c |
| Y5052 | pMKV002 pJU1064 | 1c |
| Y5285 | *bat1*∆::hphMX4 pMKV002 pJU1064 | 1c |
| Y5286 | *bat2*∆::kanMX4 pMKV002 pJU1064 | 1c |
| Y5287 | *bat2*∆::kanMX4 *bat1*∆::hphMX4 pMKV002 pJU1064 | 1c |
| YJK3851 | *bat1*∆::hphMX4 pPC10 pMKV002 | 1e |
| YJK3852 | *bat2*∆::kanMX4 pPC10 pMKV002 | 1e |
| YJK3853 | *bat1*∆::hphMX4 *bat2*∆::kanMX4 pPC10 pMKV002 | 1e, f, 2c, 3b, c, 5d, 7 |
| YJK4198 | *bat1*∆::hphMX4 *bat2*∆::kanMX4 pRS317 pPC10 YEplac195 | 2a |
| YJK4199 | *bat1*∆::hphMX4 *bat2*∆::kanMX4 pRS317 pPC10 pPC8a | 2a |
| YJK4200 | *bat1*∆::hphMX4 *bat2*∆::kanMX4 pRS317 pPC10 pPC9a | 2a |
| YJK4201 | *bat1*∆::hphMX4 *bat2*∆::kanMX4 pPC10 pRS317 pRS416 | 2a |
| YJK4203 | pPC10 pRS317 pRS416 | 2a |
| YJK4204 | pRS317 pPC10 YEplac195 | 2a |
| YJK4249 | *bat1*∆::hphMX4 *bat2*∆::kanMX4 pPC10 pRS317 pMY28 | 2a |
| YJK4250 | *bat1*∆::hphMX4 *bat2*∆::kanMX4 pPC10 pRS317 pMY22 | 2a |
| YJK4179 | *gtr1*∆::kanMX4 pPC10 pMKV002 | 2b |
| YJK4180 | *ego1*∆::kanMX4 pPC10 pMKV002 | 2b |
| YJK4166 | *ilv2*∆::natMX4 pPC10 pMKV002 | 3b, c |
| YJK4306 | *ilv3*∆::natMX4 pMKV002 pPC10 | 3b |
| YJK4307 | *ilv3*∆::natMX4 *bat1*∆::hphMX4 *bat2*∆::kanMX4 pMKV002 pPC10 | 3b |
| YJK4308 | *ilv2*∆::natMX4 *bat1*∆::hphMX4 *bat2*∆::kanMX4 pMKV002 pPC10 | 3b |
| YJK3926 | p416ADH pPC10 pRS317 | 4 |
| YJK3927 | *bat1*∆::hphMX4 *bat2*∆::kanMX4 p416ADH pPC10 pRS317 | 4 |
| YJK3928 | *bat1*∆::hphMX4 *bat2*∆::kanMX4 pPC10 pRS317 pPC2 | 4 |
| YJK3929 | *bat1*∆::hphMX4 *bat2*∆::kanMX4 pPC10 pRS317 pJK12 | 4 |
| YJK3930 | *bat1*∆::hphMX4 *bat2*∆::kanMX4 pPC10 pRS317 pJK15 | 4 |
| YJK4236 | *bat1*∆::hphMX4 *bat2*∆::kanMX4 pPC10 pRS317 pJK51 | 4a, c |
| YJK4237 | *bat1*∆::hphMX4 *bat2*∆::kanMX4 pPC10 pRS317 pJK48 | 4a, c |
| YJK4238 | *bat1*∆::hphMX4 *bat2*∆::kanMX4 pPC10 pRS317 pJK50 | 4a, c |
| YJK4239 | *bat1*∆::hphMX4 pJK47 | 5a |
| YJK4241 | *bat1*∆::hphMX4 p416ADH | 5a |
| YJK4311 | *ACO1*-GFP-kanMX4 *bat1*∆::hphMX4 p416ADH | 5b |
| YJK4312 | *ACO1*-GFP-kanMX4 *bat1*∆::hphMX4 pJK47 | 5b |
| YJK4313 | *ACO1*-GFP-kanMX4 *bat1*∆::hphMX4 pJK59 | 5b |
| YJK4385 | BY4741/BY4742 *ACO1*-VC-*HIS3*MX6/*ACO1* | 5c |
| YJK4389 | BY4741/BY4742 *ACO1*-VC-*HIS3*MX6/*ACO1 HIS3*MX6-P*_CET1_-*VN-*BAT1/BAT1* | 5c |
| YJK4391 | BY4741/BY4742 *HIS3*MX6-P*_CET1_-*VN-*BAT1/BAT1* | 5c |
| YJK4174 | *pda1*∆::kanMX4 pMKV002 pPC10 | 6a |
| YJK4175 | *lpd1*∆::kanMX4 pMKV002 pPC10 | 6a |
| YJK4269 | *pdb1*∆::kanMX4 pMKV002 pPC10 | 6a |
| YJK4270 | *lat1*∆::kanMX4 pMKV002 pPC10 | 6a |
| YJK4276 | *aco1*∆::kanMX4 pMKV002 pPC10 | 6a, c |
| YJK4277 | *pda1*∆::kanMX4 *pdb1*∆::natMX4 pMKV002 pPC10 | 6a, c |
| YJK4278 | *lat1*∆::kanMX4 *pdb1*∆::natMX4 pMKV002 pPC10 | 6a |
| YJK4363 | *snf1*∆::natMX4 pMKV002 pPC10 | 7c, d |
| YJK4364 | *bat1*∆::hphMX4 *bat2*∆::kanMX4 *snf1*∆::natMX4 pMKV002 pPC10 | 7c, d |
| YJK4367 | *reg1*∆::kanMX4 pMKV002 pPC10 | 7c, d |
| YJK4258 | *ACO1*-GFP-kanMX4 p416ADH | S2 |
| YJK4259 | *ACO1*-GFP-kanMX4 pJK47 | S2 |
| S90 | *MAT*α |  |
| S3782 | S90 parent *MAT*α *leu1*∆::hphMX4 | S1 |
| S3783 | S90 parent *MAT*α *leu9*∆::natMX4 *leu4*∆::loxP-kanMX4-loxP | S1 |
| S3806 | S90 parent *MAT*α *leu2*∆::hphMX4 | S1 |
| YJK4261 | BY4741 parent *PDB1*-GFP-*HIS3* pJK47 | S2 |
| YJK4262 | BY4741 parent *PDB1*-GFP-*HIS3* p416ADH | S2 |
| YJK4264 | BY4741 parent *LAT1*-GFP-*HIS3* p416ADH | S2 |
| YJK4265 | BY4741 parent *LAT1*-GFP-*HIS3* pJK47 | S2 |
| YJK4314 | BY4741 parent *PDB1*-GFP-*HIS3* *bat1*∆::hphMX4 p416ADH |  |
| YJK4315 | BY4741 parent *PDB1*-GFP-*HIS3* *bat1*∆::hphMX4 pJK47 |  |

^a^Unless specified, strains are isogenic with BY4742.
