# Supplementary material for: Branched-Chain Aminotransferases Control TORC1 Signaling in Saccharomyces cerevisiae
Source: PLoS Genet. 2015 Dec 11;11(12):e1005714. doi: 10.1371/journal.pgen.1005714 (PMC4684349; doi:10.1371/journal.pgen.1005714)
Supplement: S2 Table — (DOCX) [file pgen.1005714.s004.docx]

**Table S2** Plasmids used in this study.

| **Plasmid** | **Description** | **Reference/Source** |
| --- | --- | --- |
| pJK12 | *CEN6 URA3 BAT1*^K219R^ | This study |
| pJK15 | *CEN6 URA3 BAT1*^K219A^ | This study |
| pJK47 | *CEN6 URA3 BAT1-*FLAG | This study |
| pJK48 | *CEN6 URA3 BAT2*^K202A^ | This study |
| pJK50 | *CEN6 URA3 BAT2*^K202R^ | This study |
| pJK51 | *CEN6 URA3 BAT2* | This study |
| pJK59 | *CEN6 URA3 BAT1*^K219R^*-*FLAG | This study |
| pJU1064 | *CEN6 HIS3* *SCH9*^T570A^-HA_5_ | R. Loewith [[1](#_ENREF_1)] |
| pMKV002 | *CEN6 URA3 LYS2* | J. McCusker |
| pMY22 | *CEN6 URA3 TOR1* | T. Maeda [[2](#_ENREF_2)] |
| pMY28 | *CEN6 URA3 TOR1-LM* | T. Maeda [[2](#_ENREF_2)] |
| pPC2 | *CEN6 URA3 BAT1* | This study |
| pPC8a | *2µ URA3 gtr1-Q65L* (*GTR1*-GTP) *gtr2*-*S23N* (*GTR2-*GDP*)* | [[3](#_ENREF_3)] |
| pPC9a | *2µ URA3 GTR1 GTR2* | [[3](#_ENREF_3)] |
| pPC10 | *CEN6* *HIS3 HA_3_-SCH9* | [[3](#_ENREF_3)] |
| pRS317 | *CEN6 LYS2* | [[4](#_ENREF_4)] |
| pRS416 | *CEN6 URA3* | [[4](#_ENREF_4)] |
| YEplac195 | *2µ URA3* | [[5](#_ENREF_5)] |
| p416ADH | *CEN6 URA3* | [[6](#_ENREF_6)] |

**REFERENCES**

1. Binda M, Peli-Gulli MP, Bonfils G, Panchaud N, Urban J, Sturgill TW, et al. The Vam6 GEF controls TORC1 by activating the EGO complex. Mol Cell. 2009;35(5):563-73. Epub 2009/09/15. doi: 10.1016/j.molcel.2009.06.033. PubMed PMID: 19748353.

2. Takahara T, Maeda T. Transient sequestration of TORC1 into stress granules during heat stress. Molecular Cell. 2012;47(2):242-52. Epub 2012/06/26. doi: 10.1016/j.molcel.2012.05.019. PubMed PMID: 22727621.

3. Kingsbury JM, Sen ND, Maeda T, Heitman J, Cardenas ME. Endolysosomal membrane trafficking complexes drive nutrient-dependent TORC1 signaling to control cell growth in *Saccharomyces cerevisiae*. Genetics. 2014;196(4):1077-89. doi: 10.1534/genetics.114.161646. PubMed PMID: 24514902; PubMed Central PMCID: PMC3982701.

4. Sikorski RS, Hieter P. A system of shuttle vectors and yeast host strains designed for efficient manipulation of DNA in *Saccharomyces cerevisiae*. Genetics. 1989;122(1):19-27. Epub 1989/05/01. PubMed PMID: 2659436; PubMed Central PMCID: PMC1203683.

5. Gietz RD, Sugino A. New yeast-*Escherichia coli* shuttle vectors constructed with in vitro mutagenized yeast genes lacking six-base pair restriction sites. Gene. 1988;74(2):527-34. Epub 1988/12/30. PubMed PMID: 3073106.

6. Mumberg D, Muller R, Funk M. Yeast vectors for the controlled expression of heterologous proteins in different genetic backgrounds. Gene. 1995;156(1):119-22. PubMed PMID: 7737504.
