# Supplementary material for: Branched-Chain Aminotransferases Control TORC1 Signaling in Saccharomyces cerevisiae
Source: PLoS Genet. 2015 Dec 11;11(12):e1005714. doi: 10.1371/journal.pgen.1005714 (PMC4684349; doi:10.1371/journal.pgen.1005714)
Supplement: S3 Table — (DOCX) [file pgen.1005714.s005.docx]

**Table S3** Oligonucleotides used in this study.

| **Name** | **Sequence (5’-3’)^ab^** | **Purpose** |
| --- | --- | --- |
| JK5 | GTATTCTGGGCCTCCATGTC | Confirmation of gene disruption by MX4 cassette |
| JK6 | GACATCATCTGCCCAGATGC | Confirmation of gene disruption by MX4 cassette |
| JK44 | AGCAACAGACTATGCTACAAGAGCTTGGCCAGGTGGTGTTGGCGACAAAAGATTGGGTGCTAACTATGCCCCATGCATCT | *BAT1*^K219R^ cloning |
| JK45 | AGCAACAGACTATGCTACAAGAGCTTGGCCAGGTGGTGTTGGCGACAAAGCTTTGGGTGCTAACTATGCCCCATGCATCT | *BAT1*^K219A^ cloning |
| JK46 | CAATCTTTTGTCGCCAACACCACCTG | *BAT1*^K219R^ cloning |
| JK47 | CAAAGCTTTGTCGCCAACACCACCTG | *BAT1*^K219A^ cloning |
| JK222 | TGAGCTAAGAGGAGATAAATACAACAGAATCAATTTTCAACAGCTGAAGCTTCGTACGC | *ILV2* disruption with MX4 cassette |
| JK223 | TTTTTACTGAAAATGCTTTTGAAATAAATGTTTTTGAAATGCATAGGCCACTAGTGGATCTG | *ILV2* disruption with MX4 cassette |
| JK224 | TGGAGGAGATATTCATAGTG | Confirmation of *ILV2* disruption |
| JK225 | GTCAGATCAGACACACCAAC | Confirmation of *ILV2* disruption |
| JK226 | TTCTTGTATTTTTTTGTAAACAGCCAAGAAAAAAGTAGAGCAGCTGAAGCTTCGTACGC | *ILV3* disruption with MX4 cassette |
| JK227 | ATCTCTATATATATATTCATCGATTGGGGCCTATAATGCAGCATAGGCCACTAGTGGATCTG | *ILV3* disruption with MX4 cassette |
| JK228 | GAGAGATCAGATTGCTCTTC | Confirmation of *ILV3* disruption |
| JK229 | GCTTCGTCGTATATTGGATG | Confirmation of *ILV3* disruption |
| JK341 | GTTGGACACTCTGTCTGAAG | Confirmation of *PDA1* disruption |
| JK342 | AAGAACCTCTACAGCAGAGG | Confirmation of *PDA1* disruption |
| JK343 | TGTGTTTGCGTAATCCTGTC | Confirmation of *LPD1* disruption |
| JK344 | GTAGTCCTGGTGAGCAAAAG | Confirmation of *LPD1* disruption |
| JK369 | CCAGGAGGCTGTGGTGACAAGAGGCTAGGTGCAAACTACG | *BAT2*^K202R^ cloning |
| JK370 | CGTAGTTTGCACCTAGCCTCTTGTCACCACAGCCTCCTGG | *BAT2*^K202R^ cloning |
| JK371 | CCAGGAGGCTGTGGTGACAAGGCTCTAGGTGCAAACTACG | *BAT2*^K202A^ cloning |
| JK372 | CGTAGTTTGCACCTAGAGCCTTGTCACCACAGCCTCCTGG | *BAT2*^K202A^ cloning |
| JK376 | GGGGGATCCTTACTTATCGTCGTCATCCTTGTAATCGTTCAAGTCGGCAACAGTTT | *BAT1-*FLAG cloning |
| JK377 | TAAAGTTTATTTACAAGATAACAAAGAAACTCCCTTAAGCCAGCTGAAGCTTCGTACGC | *ARO10* disruption with MX4 cassette |
| JK378 | ACAATTGGTAGCAGTGTTTTATAATTGCGCCCACAAGTTTGCATAGGCCACTAGTGGATCTG | *ARO10* disruption with MX4 cassette |
| JK413 | CCATCGTCAATTACGCAGAC | Confirmation of *ACO1* disruption |
| JK414 | GTCGGATTGCCCTTTTAAGC | Confirmation of *ACO1* disruption |
| JK415 | AGAAGCACCTAGCCTCTTTC | Confirmation of *PDB1* disruption |
| JK416 | GCTCTTTCGCTTAGGTTCCA | Confirmation of *PDB1* disruption |
| JK417 | AGATCCTGGGTTCTGGATAC | Confirmation of *LAT1* disruption |
| JK418 | CTCTCTCACCCAAGTTGTAT | Confirmation of *LAT1* disruption |
| JK421 | TGGTTCTGCCTTAAATAAAATTAAGGCCGATGAGAAGAAAGGTCGACGGATCCCCGGGTT | *ACO1*-GFP construction |
| JK422 | AATAAATAATAACCTTTAAAAGATTATAACAATGTTTTCATCGATGAATTCGAGCTCGTT | *ACO1*-GFP construction |
| JK423 | GGTCCATGGTTGAAATACAG | *ACO1*-GFP confirmation |
| JK424 | CCTTGTCCCTTATTCTGCTG | *PDB1*-GFP confirmation |
| JK425 | ACGACAAGTACAAACTGTCC | *LAT1*-GFP confirmation |
| JK426 | TTTAGTAAGTTCTTTTACTTTTGCAATAATTTTGTTCAACCAGCTGAAGCTTCGTACGC | *PDB1* disruption with MX4 cassette |
| JK427 | ATCTCCTTCTTTCTCTCCTTCCTATTGGATTGAAGTTTATGCATAGGCCACTAGTGGATCTG | *PDB1* disruption with MX4 cassette |
| JK428 |  |  |
| JK467 | GAAGTTTTTTTTTGTAACAAGTTTTGCTACACTCCCTTAATAAAGTCAACCAGCTGAAGCTTCGTACGC | *SNF1* disruption with MX4 cassette |
| JK468 | CGATACATAAAAAAAAGGGAACTTCCATATCATTCTTTTACGTTCCACCAGCATAGGCCACTAGTGGATCTG | *SNF1* disruption with MX4 cassette |
| JK469 | AGGCTATGATGTCCCATATG | Confirmation of *SNF1* disruption |
| JK470 | TGTTCTGGCAGCATGATTTG | Confirmation of *SNF1* disruption |
| JK489 | CAGTGGATATTGAAGGAAGG | Confirmation of *REG1* disruption |
| JK490 | GAGGTGTTTCGCTTATTGAC | Confirmation of *REG1* disruption |
| JK532 | GGTACCGGCCTTTAAGCTTTGAAAAAAAAAAAGCATCTGAAAAAAGAATTCGAGCTCGTTTAAAC | *HIS3*MX6-P*_CET1_*-VN-*BAT1* construction |
| JK533 | TCTGATGGAGAATTTCCCCAACTTCAAGGAATGTCTCTGCAACATagtaccaccagaaccCTCGATGTTGTGGCGGATC | *HIS3*MX6-P*_CET1_*-VN-*BAT1* construction |
| JK534 | TGGTTCTGCCTTAAATAAAATTAAGGCCGATGAGAAGAAAGGTCGACGGATCCCCGGGTT | *ACO1*-VC-*HIS3*MX6 construction |
| JK535 | AATAAATAATAACCTTTAAAAGATTATAACAATGTTTTCATCGATGAATTCGAGCTCGTT | *ACO1*-VC-*HIS3*MX6 construction |
| JK539 | AGggttctggtggtactATG | *HIS3*MX4-P*_CET1_*-VN confirmation |
| JK540 | AACCCGGGGATCCGTCGACC | VC/VD-*HIS3*MX6 confirmation |
| JK541 | AACGAGCTCGAATTCATCGA | VC/VD-*HIS3*MX6 confirmation |
| MPDC01 | TGCTCTAGAATGTTGCAGAGACATTCC | *BAT1* cloning into p416ADH |
| MPDC02 | CGCGGATCCTTAGTTCAAGTCGGCAAC | *BAT1* cloning into p416ADH |
| MPDC10 | GCATAGTTAGCACCCAATTT | P*_CET1_-*VN*-BAT1* confirmation |
| MPDC13 | TGCTCTAGAATGACCTTGGCACCCCTAG | *BAT2* cloning into p416ADH |
| MPDC14 | CGCGGATCCTCAGTTCAAATCAGTAACAAC | *BAT2* cloning into p416ADH |
| NDMC92 | TATAAACGCAAAATCAGCTAGAACCTTAGCATACTAAAACCGGATCCCCGGGTTAATTAA | *BAT1* disruption with MX4 cassette |
| NDMC93 | TTTTTGGGGGGGGAGGGGATGTTTACCTTCATTATCAGAATTCGAGCTCGTTTAAAC | *BAT1* disruption with MX4 cassette |
| NDMC94 | ATGTTAACGCTGGATAAG | Confirmation of *BAT1* disruption |
| NDMC95 | AAAGTCCAGCGAGATACC | Confirmation of *BAT1* disruption |
| NDMC96 | ATACCACTATATACGTGTACG | Confirmation of *BAT2* disruption |
| NDMC97 | AGGCCAGCACTAGATGAC | Confirmation of *BAT2* disruption |
| NDMC98 | ATATTTGACTTCGACGTG | Confirmation of *THI3* disruption |
| NDMC100 | TACAACAACGTCTTAGCG | Confirmation of *ARO10* disruption |
| NDMC101 | AGAGCCAATGATTTCGTC | Confirmation of *ARO10* disruption |

^a^ Underlined sequence is homologous to vector or cassette sequence

^b^ lowercase is linker sequence
