# Supplementary material for: Branched-Chain Aminotransferases Control TORC1 Signaling in Saccharomyces cerevisiae
Source: PLoS Genet. 2015 Dec 11;11(12):e1005714. doi: 10.1371/journal.pgen.1005714 (PMC4684349; doi:10.1371/journal.pgen.1005714)
Supplement: S4 Table — Average metabolite levels from cell extracts prepared from cultures grown in SD+ile leu val gln (T0 control), rapamycin treatment (200 nM, 30 min, +Rapa), 2 hr leucine starvation (-Leu), and addition for 1 hr following leucine starvation of leucine, glutamine, or d-KG, as indicated. Extracts were prepared in triplicate for amino acids and organic acids, and duplicate for acetyl CoA. (DOCX) [file pgen.1005714.s006.docx]

**Table S4.** Relative amounts of metabolite abundance in WT and *bat1 bat2* yeast strains. Average metabolite levels from cell extracts prepared from cultures grown in SD+ile leu val gln (T0 control), rapamycin treatment (200 nM, 30 min, +Rapa), 2 hr leucine starvation (-Leu), and addition for 1 hr following leucine starvation of leucine, glutamine, or d-KG, as indicated. Extracts were prepared in triplicate for amino acids and organic acids, and duplicate for acetyl CoA.

|  |  | **WT** | | | | | | ***bat1 bat2*** | | | | | |
| --- | --- | --- | --- | --- | --- | --- | --- | --- | --- | --- | --- | --- | --- |
| **Metabolite** | **units** | **control** | **+rapa** | **-leu** | **+leu** | **+glu** | **+dKG** | **control** | **+rapa** | **-leu** | **+leu** | **+glu** | **+dKG** |
| Lactate | µM | 29.9321 | 32.3991 | 29.0071 | 33.4907 | 27.0517 | 30.1576 | 30.4134 | 29.3198 | 27.5202 | 31.6506 | 28.4461 | 28.0660 |
| Pyruvate | µM | 26.8241 | 24.0584 | 18.4987 | 17.8271 | 13.5672 | 12.9834 | 15.7141 | 17.2102 | 11.5846 | 11.1926 | 12.2359 | 11.0675 |
| Succinate | µM | 70.4668 | 3.5368 | 1.3449 | 7.0588 | 2.5613 | 4.5380 | 50.6406 | 13.5399 | 10.3218 | 19.3352 | 13.5666 | 28.1003 |
| Fumarate | µM | 5.0543 | 1.3162 | 0.8283 | 2.7651 | 0.8425 | 0.9790 | 2.8233 | 2.1460 | 2.2808 | 3.1943 | 1.9653 | 1.6921 |
| Malate | µM | 12.6103 | 2.1325 | 0.7104 | 7.5240 | 0.9092 | 1.4896 | 10.1222 | 5.9040 | 7.4468 | 12.1189 | 6.1572 | 4.8059 |
| α-ketoglutarate | µM | 13.1830 | 5.0016 | 4.0767 | 5.7041 | 4.0513 | 5.3616 | 9.2448 | 6.7013 | 2.3740 | 1.5706 | 3.6396 | 5.6954 |
| Citrate | µM | 18.0641 | 23.6521 | 38.5426 | 20.8124 | 45.0329 | 47.4020 | 11.9489 | 18.1272 | 13.2993 | 22.8899 | 11.2170 | 12.2567 |
| Acetyl CoA | pM/500µl | 763.6551 | 582.7607 | 691.1461 | 545.6240 | 512.1001 | 378.6768 | 309.1085 | 338.2634 | 380.7773 | 501.0935 | 342.8004 | 368.5104 |
| Glycine | nM/100µl | 11.4800 | 10.8000 | 7.1633 | 12.4633 | 6.8333 | 6.3833 | 8.4267 | 7.3767 | 7.9633 | 8.0633 | 7.4000 | 7.9267 |
| Alanine | nM/100µl | 18.8500 | 48.1633 | 76.4567 | 9.3700 | 71.6500 | 64.8267 | 26.6933 | 33.6000 | 68.2033 | 40.8567 | 61.3567 | 53.1633 |
| Serine | nM/100µl | 14.3033 | 4.0933 | 6.9800 | 7.2000 | 6.7800 | 6.3933 | 5.3433 | 2.4600 | 2.8933 | 2.5167 | 3.3467 | 3.0867 |
| Proline | nM/100µl | 5.2933 | 3.8700 | 9.7300 | 2.5700 | 9.8300 | 9.9100 | 2.4567 | 2.3600 | 1.9367 | 1.5033 | 2.3567 | 2.6533 |
| Valine | nM/100µl | 6.1500 | 14.7567 | 5.3100 | 12.2100 | 4.8467 | 4.4767 | 10.5700 | 11.9400 | 29.5133 | 21.7633 | 26.9400 | 31.0633 |
| Leucine/Isoleucine | nM/100µl | 2.4667 | 10.6567 | 1.8733 | 27.9967 | 1.5700 | 1.5333 | 2.5000 | 4.1767 | 3.2133 | 13.2200 | 2.7867 | 3.1433 |
| Methionine | nM/100µl | 0.1067 | 0.2333 | 0.0833 | 0.0100 | 0.0500 | 0.0733 | 0.1033 | 0.1267 | 0.0233 | 0.0300 | 0.0400 | 0.0700 |
| Histidine | nM/100µl | 3.0933 | 2.7333 | 4.1733 | 3.1333 | 3.9667 | 4.3867 | 2.0000 | 1.6967 | 1.6067 | 1.2333 | 1.4233 | 1.3700 |
| Phenylalanine | nM/100µl | 0.3867 | 0.6700 | 1.3400 | 0.3567 | 1.2467 | 1.1700 | 0.6167 | 0.6100 | 0.6200 | 0.3300 | 0.6267 | 0.4400 |
| Tyrosine | nM/100µl | 0.3033 | 0.4933 | 3.3400 | 0.5233 | 3.8600 | 3.5133 | 0.6500 | 0.7300 | 0.7967 | 0.3167 | 0.8633 | 0.6300 |
| Asparagine/Aspartic acid | nM/100µl | 12.5867 | 35.7700 | 17.5267 | 10.9467 | 14.5867 | 11.7433 | 8.7567 | 13.0933 | 9.0900 | 6.6167 | 5.7600 | 3.5133 |
| Glutamine/Glutamate | nM/100µl | 151.8200 | 175.8000 | 68.2367 | 61.3800 | 69.2933 | 71.7833 | 92.9300 | 95.9833 | 30.5467 | 33.6300 | 41.1000 | 47.5667 |
| Ornithine | nM/100µl | 4.1433 | 4.6633 | 4.6733 | 3.5000 | 3.6100 | 4.1533 | 1.2700 | 1.3500 | 1.0467 | 1.1700 | 1.2167 | 0.9733 |
| Citrulline | nM/100µl | 5.7467 | 5.5400 | 3.1700 | 2.7800 | 2.9400 | 2.8967 | 0.5233 | 0.4800 | 0.2567 | 0.2633 | 0.2200 | 0.2267 |
| Arginine | nM/100µl | 5.4833 | 5.7700 | 10.3733 | 7.8900 | 12.1167 | 13.3867 | 2.9900 | 3.1133 | 3.0667 | 2.9433 | 3.1967 | 2.8867 |
